# Association between *OPG* polymorphisms and osteoporosis risk: An updated meta-analysis

**DOI:** 10.3389/fgene.2022.1032110

**Published:** 2022-11-09

**Authors:** Xu Han, Lai Zheng, Yi-Yang Mu, Hong-Zhuo Li, Xiao-Feng He

**Affiliations:** ^1^ Heping Hospital Affiliated to Changzhi Medical College, Changzhi, Shanxi, China; ^2^ Department of Orthopaedics, Heping Hospital Affiliated to Changzhi Medical College, Changzhi, Shanxi, China; ^3^ Institute of Evidence-Based Medicine, Heping Hospital Affiliated to Changzhi Medical College, Changzhi, Shanxi, China; ^4^ Department of Epidemiology, School of Public Health, Southern Medical University, Guangzhou, China

**Keywords:** osteoprotegerin, OPG, polymorphism, meta-analysis, BFDP, FPRP

## Abstract

**Background:** Numerous studies have demonstrated an association between osteoprotegerin (*OPG*) polymorphisms (A163G (rs3102735), T245G (rs3134069), T950C (rs2073617), G1181C (rs2073618)) and osteoporosis risk. However, their conclusions are inconsistent. In addition, some new studies have been updated, and more importantly, previous meta-analyses have not tested for false-positive results. In order to further explore these associations, we recently conducted a meta-analysis.

**Objectives:** To study the relationship between *OPG* polymorphisms A163G, T245G, T950C, G1181C and the risk of osteoporosis.

**Methods:** PubMed, Medline, International Statistical Institute (ISI), China National Knowledge Infrastructure (CNKI) and China Wanfang Database were used for research searches. Associations were assessed with five genetic models using odds ratios (ORs) with 95% confidence intervals (CIs). In addition, confidence in statistically significant associations was assessed using false-positive report probability (FPRP), Bayesian probability of False discovery (BFDP), and Venice criteria.

**Results:** On the whole, the *OPG* A163G polymorphism was not significantly associated with risk of osteoporosis. However, in a subgroup analysis, we found that the *OPG* A163G polymorphism increased the risk of osteoporosis in Caucasians (AG + GG vs AA: OR = 1.35, 95% CI = 1.06–1.73; AA + GG vs AG: OR = 0.64, 95% CI = 0.49–0.82) and the female (G vs A: OR = 1.30, 95% CI = 1.03–1.64; AG + GG vs AA: OR = 1.42, 95% CI = 1.18–1.71). At the same time, the *OPG* G1181C polymorphism reduces the risk of osteoporosis (C vs G: OR = 0.84, 95% CI = 0.74–0.95; CC vs GG: OR = 0.75, 95% CI = 0.60–0.93; GC + CC vs GG: OR = 0.80, 95% CI = 0.67–0.95; CC vs GG + GC: OR = 0.84, 95% CI = 0.70–1.00). Moreover, a significantly decreased risk of osteoporosis was also discovered in Asian (C vs G: OR = 0.80, 95% CI = 0.66–0.98; CC vs GG: OR = 0.67, 95% CI = 0.47–0.95; GC + CC vs GG: OR = 0.74, 95% CI = 0.58–0.95) and the female (C vs G: OR = 0.85, 95% CI = 0.75–0.97; CC vs GG: OR = 0.77, 95% CI = 0.61–0.96; GC + CC vs GG: OR = 0.79, 95% CI = 0.66–0.95). Finally, we did not find a close association between *OPG* T245G and T950C polymorphisms and osteoporosis risk. However, when we retained only studies in the control group that was consistent with Hardy-Weinberg equilibrium (HWE) and high-quality scores, we observed that the *OPG* A163G polymorphism increased the risk of osteoporosis in the overall analysis (G vs A: OR = 1.40, 95% CI = 1.16–1.68; GG vs AA: OR = 1.96, 95% CI = 1.20–3.21; AG + GG vs AA: OR = 1.45, 95% CI = 1.22–1.72). Finally, after the credibility assessment, we concluded that all statistically significant association results in the meta-analysis in this study and those in the previous study were ‘positive results with low confidence’.

**Conclusion:** In conclusion, our study concluded that all meaningful results between *OPG* A163G and G1181C polymorphisms and osteoporosis risk were false-positive results rather than true associations.

## Introduction

Osteoporosis is a systemic bone disease characterized by deterioration of femoral microstructure and low bone mass, which increases fracture susceptibility and bone fragility (Rachner et al., 2011). The World Health Organization has a specific definition of osteoporosis: bone mineral density is measured by dual energy X-ray (DEXA), and the obtained bone mineral density value is called T-score. The T-score is more than 2.5 standard deviations below the average of the normal population (young healthy people with peak bone mass), and osteoporosis can be diagnosed. (Genant et al., 1999). Osteoporosis affects about 200 million people worldwide, which affects about 12% of men and 30% of women. Once osteoporosis is present, there may be significant pain and deformities, which also increase morbidity and mortality (Lin and Lane, 2004; Uzzan et al., 2007; Pisani et al., 2016). In addition, osteoporosis increases the risk of various diseases, such as fractures and pneumonia (Rapp et al., 2015), and around nine million people worldwide suffer fractures every year because of osteoporosis (Compston et al., 2019). In China, about 2.3 million people suffered fractures due to osteoporosis in 2010, and at this rate, the number of fractures caused by osteoporosis will exceed six million by 2050 (Si et al., 2015), which will undoubtedly increase the burden on families and the healthcare environment as a whole. From the above, we can understand that osteoporosis has become an inescapable health problem. Therefore, we must explore which risk factors contribute to osteoporosis.

The risk of osteoporosis includes environmental factors and genetic factors. The main environmental factors are changes in living habits, including smoking, drinking and improper exercise (Ng et al., 2006; Kaufman et al., 2008; Binici and Gunes, 2010). With the development of medical treatment, many studies have shown that genetic factors play an important role in the pathogenesis. (Ralston, 2001; Recker and Deng, 2002). The heritability of osteoporosis-related traits is estimated to be as high as 60–80% (Uitterlinden et al., 2004). So far, many risk genes have been found for osteoporosis. Among these risk genes, *ITGA1*, *ESR1*, *SPP1*, *LRP4*, and *LRP5* have been confirmed to be related to bone matrix composition, bone mineral density (BMD) homeostasis and bone remodeling, thereby affecting BMD (Prestwood et al., 1995), which can lead to osteoporosis. In addition, there are many candidate genes (*VDR*, *TGFB1*, *COL1A1*, and *OPG*), but the association of these polymorphisms with osteoporosis risk has not been thoroughly demonstrated (Prestwood et al., 1995; Jørgensen et al., 2012; Saccone et al., 2015; Tsukasaki et al., 2020).

Osteoprotegerin (*OPG*), also called osteoclast genesis inhibitory factor, is a secreted glycoprotein discovered by Simonet et al., in 1997. It is a single copy gene cluster located on human chromosome 8q24.2. So far, many researches have shown that *OPG* gene has relationship to the pathogenesis of osteoporosis (Chung et al., 2003; Yu et al., 2013; Sun et al., 2014). The main mechanism is to inhibit osteoclast differentiation and maturation through the signal transduction system *OPG*/*RANKL*/*RANK*. The gene *OPG* can competitively bind *RANK* on the cell membrane surface of osteoclasts. Moreover, its binding ability is stronger than *RANKL* (Simonet et al., 1997), which can effectively interfere with the combination of *RANKL* and *RANK*. Common *OPG* polymorphisms associated with osteoporosis risk include A163G (rs3102735), T950C (rs2073617), and T245G (rs3134069), which are located at the promoter of the gene, while G1181C (rs2073618) is located at the first exon of the gene. Although researchers have published meta-analyses of *OPG* and osteoporosis risk. (Guo et al., 2014; Luo et al., 2014; Li et al., 2017; Li et al., 2021), but the results of studies on these gene polymorphisms are not completely consistent or even conflicting. The reasons for these different results are as follows: first of all, the number of samples is small and the included research literature is less; second, some studies have not carried out literature quality evaluation, which may lead to bias in the final results if low-quality literature is not excluded; at the same time, heterogeneity of selected literature, study design of variables, and whether or not to perform HWE tests also affect the results of meta-analysis. Finally, previously published meta-analyses have never evaluated positive results to determine multiple comparisons. In order to prove the relationship between the two, we need to provide more accurate and reliable theoretical evidence, so this is where we did an updated meta-analysis.

## Materials and methods

This study was conducted according to the statement on preferred reporting items for systematic reviews and meta-analyses (Supplementary Table S5 PRISMA Checklist) (Moher et al., 2009).

### Search strategy

The literature was searched using PubMed, Medline, International Statistical Institute (ISI), China National Knowledge Infrastructure (CNKI) and China Wanfang Database. The retrieval strategy was as follows (‘mutation’ OR ‘polymorphism’ OR ‘variation’ OR ‘variant’ OR ‘SNP’ OR ‘genetic association study’ OR ‘genome-wide association study’ OR ‘genotype’ OR ‘allele ‘) AND (osteoporosis OR osteoporoses) AND (*OPG* OR osteoprotegerin). The literature search was conducted until 20 March 2022. In addition, we carefully reviewed each reference list for meta-analyses in the literature to identify all eligible studies.

### Selection criteria

The inclusion criteria were as follows: 1) to describe the relationship between *OPG* A163G, T245G, T950C and G1181C polymorphisms and the risk of osteoporosis (including the ethnic or demographic composition of the study subjects, the sex of the study subjects, and the genotyping technique used, Supplementary Table S2); 2) case-control study or cohort study, and all patients met the diagnostic criteria for osteoporosis; 3) detailed genotype data or odds ratio (OR) with 95% confidence interval (CI) could be extracted from the literature. Exclusion criteria were as follows: 1) not a case-control or cohort study; 2) failure to provide genotype data or duplicate genotype data; 3) meta-analyses, reviews, letters, case reports, and only including abstracts.

### Data extraction and quality score assessment

We designed the data extraction form in advance. All literatures were screened according to the inclusion criteria and exclusion criteria, and then the literature data were extracted for statistical analysis. When the two authors obtained different results and still could not reach a consensus after discussion, the third author extracted the data again, and finally the three authors jointly checked and confirmed. If the data in the article is unclear or disputed, we will contact the original author to obtain the original data. The extracted information included: year of publication, first author of the article, country of study, ethnicity of study subjects, sex of study subjects, female menopause, polymorphism genotyping technique, type of control group, sample size and mean age for case and control groups, and quality score of all articles (Supplementary Table S2).

The quality assessment for all included articles was assessed separately by two authors. We referenced and improved the specific criteria for quality scores from previous studies (Guo et al., 2014; Luo et al., 2014; Li et al., 2017; Li et al., 2021). The full score of our reformulated quality assessment scale is 20 points, with more than 12 points as excellent (including) and less than 12 points as poor. The specific criteria of the quality evaluation scale are shown in Supplementary Table S3, and the score of each included paper is shown in Supplementary Table S4.

### Statistical analysis

The correlation between *OPG* gene polymorphisms (A163G, T245G, T950C, and G1181C) and osteoporosis was expressed by ORs and corresponding 95% confidence intervals. The following five genetic models were selected for evaluation: 1) allele model (variant allele vs wildtype allele); 2) dominant mode (homozygous variant + heterozygous variant vs homozygous wildtype); 3) recessive model (homozygous variant vs heterozygous variant + homozygous wild type); 4) over-dominant model (homozygous wild type + homozygous variant vs heterozygous variant); 5) additive model (homozygous variant vs homozygous wild type). Chi-square q test and I^2^ value were used to evaluate the heterogeneity of the results. When *p* was less than 0.10 and/or *I* ^2^ was greater than 50% (Li et al., 2005), the random effects model (MANTEL and HAENSZEL, 1959) is selected. If not, the fixed effects model is adopted (DerSimonian and Laird, 2015), and the goodness-of-fit test is further carried out to test the applicability of the model (Chen et al., 2015). At the same time, meta-regression was used to try to find the source of heterogeneity. Subgroup analyses were then performed according to ethnicity, sex, female menopause and control type. Two methods were used in the sensitivity analysis: 1) each study was excluded separately; 2) Only studies that met both high quality scores and HWE were retained. Prior to this, the control group was tested for HWE compliance using chi-square goodness of fit. *p* > 0.05 was defined as HWE, *p* < 0.05 is defined as HWD. Begg’s funnel plot (Begg and Mazumdar, 1994) and Egger’s test (Egger et al., 1997) were used to evaluate the existence of publication bias. If publication bias exists, the number of missing studies is estimated and supplemented using a non-parametric “trim and fill” approach (Dual and Tweedie, 2000). False-positive report probability (FPRP) (Wacholder et al., 2004), Bayesian False Discovery probability (BFDP) (Wakefield, 2007), and Venice criterion (Ioannidis et al., 2008) were used to assess the confidence of statistically significant associations. Stata 15.0 software was used to calculate all statistical analyses.

## Results

### Study characteristics

**FIGURE 1:**
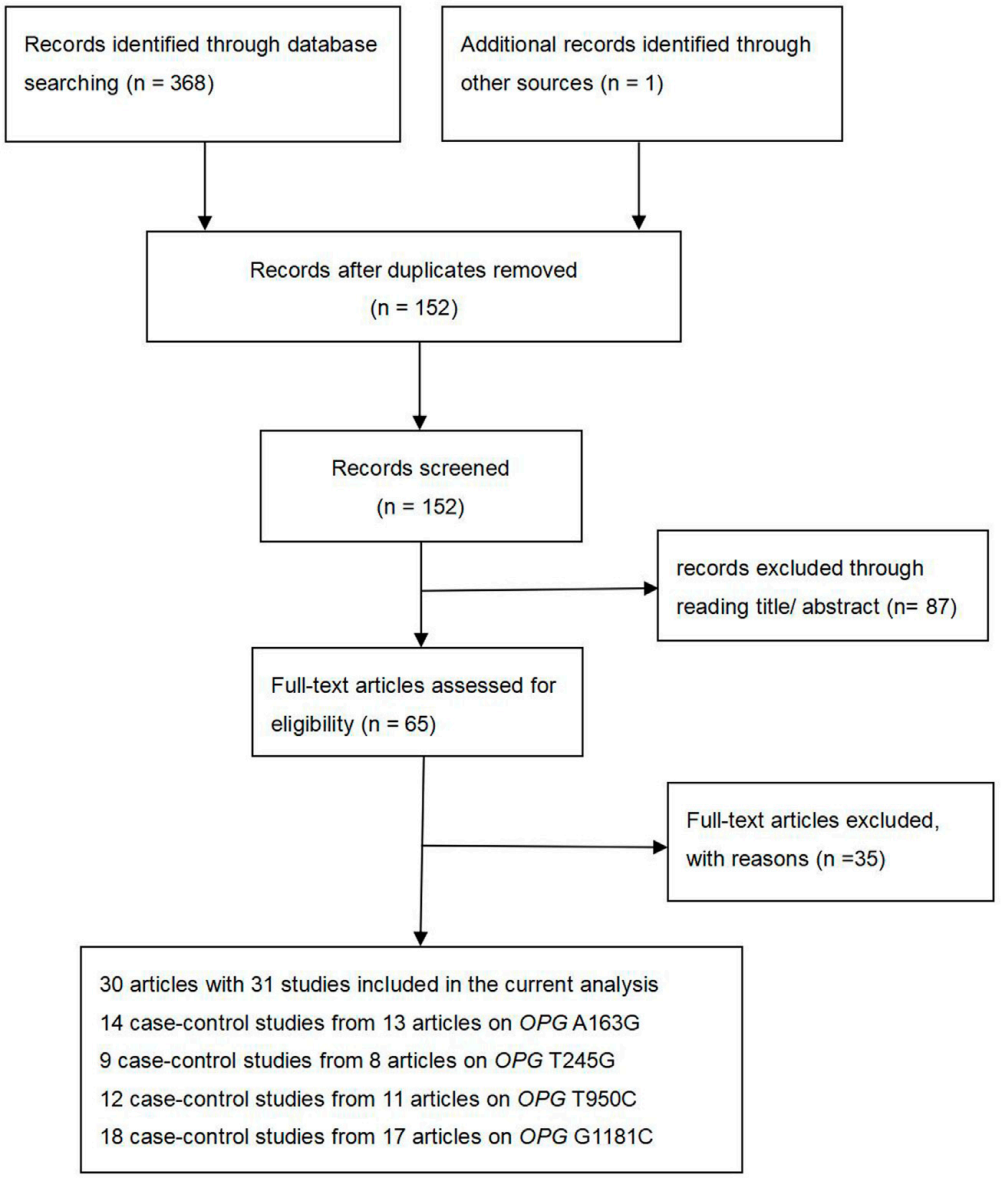
Flow diagram of the literature search.

At the beginning of the period, 369 articles were searched from ISI, PubMed, Medline, CNKI and the China Wan-fang databases. There are 152 records left after duplicates are removed. After careful screening for titles and abstracts, we excluded 87 papers. In addition, 17 items were excluded because of data duplication or the inability to extract detailed data, and 18 items were excluded because of inadequate controls. Finally, 30 articles and 31 studies were included in our study (Supplementary Table S1). A total of 31 studies (Figure 1) were included in our study (including 8,402 osteoporosis cases and 7,517 controls), of which 14 studies reported *OPG* A163G (2,379 cases and 2,229 controls), nine studies investigated *OPG* T245G (941 cases and 1,019 controls), 12 studies investigated *OPG* T950C (1,610 cases and 1,234 controls), and 18 studies reported *OPG* G1181C (3,472 cases and 3,035 controls), as shown in Supplementary Table 1. In addition, *OPG* A163G had three low-quality studies and 11 high-quality articles, *OPG* T245G had two low-quality articles and seven high-quality articles, *OPG* T950C had five low-quality articles and seven high-quality articles, and *OPG* G1181C had two low-quality articles and 16 high-quality articles. The complete characteristics and genotype frequencies of the final included literatures are shown in Supplementary Table 1.

### Quantitative synthesis

**FIGURE 2:**
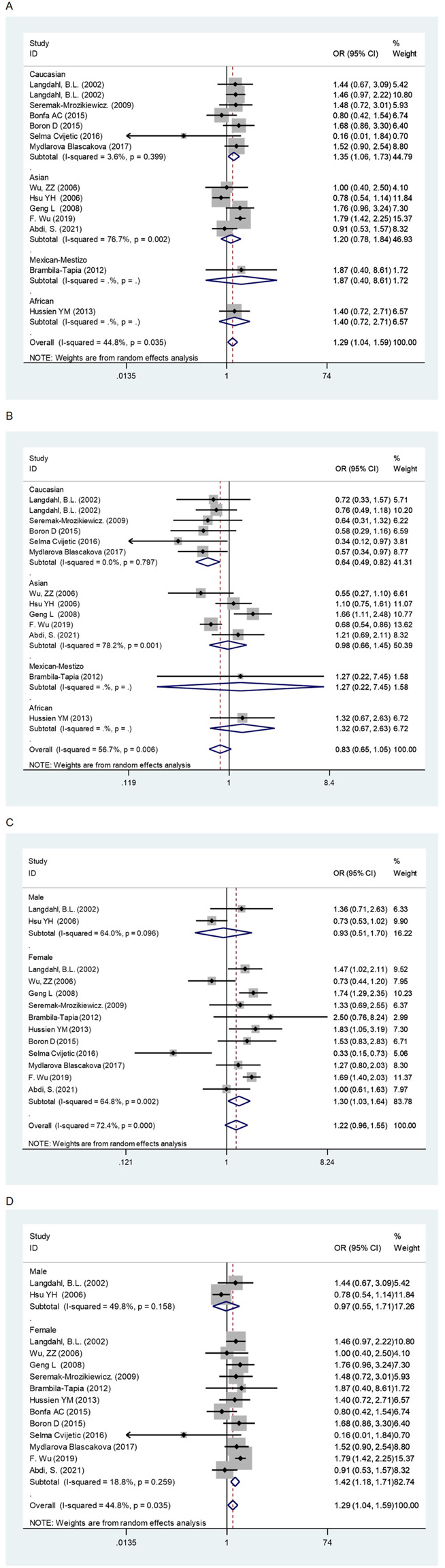
The forest plots of all selected studies on the adjusted association between the OPG A163G polymorphism and risk of osteoporosis in overall and subgroup analyses by ethnicity and sex (A: AG + GG vs AA; **(B)** AA + GG vs AG; **(C)** G vs A; **(D)** AG + GG vs AA).

In the overall analysis, no meaningful association was found between the *OPG* A163G polymorphism and the risk of osteoporosis. However, in the following ethnic subgroup analysis, we found that the *OPG* A163G polymorphism increased the risk of osteoporosis in Caucasians (AG + GG vs AA: OR = 1.35, 95% CI = 1.06–1.73; AA + GG vs AG: OR = 0.64, 95% CI = 0.49–0.82, Table 1 and Figure 2). Unfortunately, in spite of a significant association in the African population, the sample size was only one study, which may limit the statistical power of this finding and thus we do not consider it sufficient to state a significant association between the two populations in the African population. In the sex subgroup, the *OPG* A163G polymorphism can significantly increase the risk of osteoporosis in females (G vs A: OR = 1.30, 95% CI = 1.03–1.64; AG + GG vs AA: OR = 1.42, 95% CI = 1.18–1.71, Table 1 and Figure 2). Similarly, in the female menopause subgroup, the *OPG* A163G polymorphism also increases the risk of osteoporosis in both non-postmenopausal women (G vs A: OR = 1.68, 95% CI = 1.36–2.07; GG vs AA: OR = 2.74, 95% CI = 1.64–4.58; AG + GG vs AA: OR = 1.37, 95% CI = 1.04–1.79; GG vs AA + AG: OR = 2.31, 95% CI = 1.55–3.43, Table 1 and Figure 2) and postmenopausal women (AG + GG vs AA: OR = 1.40, 95% CI = 1.06–1.85; GG vs AA + AG: OR = 0.67, 95% CI = 0.54–0.82, Table 1 and Figure 2).

**TABLE 1:** Meta-analysis of the association of OPG A163G polymorphism with risk of osteoporosis.

Variable	n (Cases/Controls)	G vs A	GG vs AA	AG + GG vs AA	GG vs AA + AG	AA + GG vs AG					
		OR (95%CI)	*Ph/I* ^*2*^ (%)	OR (95%CI)	*Ph/I* ^*2*^ (%)	OR (95%CI)	*Ph/I* ^*2*^ (%)	OR (95%CI)	*Ph/I* ^*2*^ (%)	OR (95%CI)	*Ph/I* ^*2*^ (%)
Overall	14 (2,379/2,229)	1.22 (0.96–1.55)	<0.001/72.4	1.38 (0.75–2.53)	0.002/61.1	**1.29 (1.04–1.59)**	0.035/44.8	1.16 (0.65–2.09)	<0.001/71.5	0.83 (0.65–1.05)	0.006/56.7
Ethnicity											
Caucasian	7 (923/804)	1.17 (0.82–1.67)	0.036/58.0	0.82 (0.29–2.34)	0.163/36.5	**1.35 (1.06–1.73)**	0.399/3.6	0.69 (0.25–1.89)	0.074/50.2	**0.64 (0.49–0.82)**	0.797/0.0
Asian	5 (1,297/1,345)	1.13 (0.76–1.67)	<0.001/86.2	1.35 (0.59–3.10)	0.001/77.4	1.20 (0.78–1.84)	0.002/76.7	1.18 (0.56–2.49)	<0.001/81.9	0.98 (0.66–1.45)	0.001/78.2
Mexican-Mestizo	1 (9/30)	2.50 (0.76–8.24)	NA	8.40 (0.63–3.10)	NA	1.87 (0.40–8.61)	NA	8.29 (0.65–104.89)	NA	1.27 (0.22–7.45)	NA
African	1 (150/50)	**1.83(1.05–3.19)**	NA	**9.14 (1.19–70.41)**	NA	1.40 (0.72–2.71)	NA	**9.33 (1.23–70.88)**	NA	1.32 (0.67–2.63)	NA
Sex											
Male	2 (336/362)	0.93 (0.51–1.70)	0.096/64.0	0.55 (0.11–2.77)	0.150/51.7	0.97 (0.55–1.71)	0.158/49.8	0.53 (0.12–2.25)	0.185/43.1	1.01 (0.72–1.43)	0.339/0.0
Female	12 (2043/1867)	**1.30 (1.03–1.64)**	0.002/64.8	1.72 (0.97–3.07)	0.030/49.9	**1.42 (1.18–1.71)**	0.259/18.8	1.34 (0.72–2.46)	<0.001/70.7	0.80 (0.61–1.05)	0.005/56.7
Female menopause											
NP	5 (611/669)	**1.68 (1.36–2.07)**	0.775/0.0	**2.74 (1.64–4.58)**	0.498/0.0	**1.37 (1.04–1.79)**	0.475/0.0	**2.31 (1.55–3.43)**	0.377/3.0	1.18 (0.75–1.87)	0.083/55.1
*p*	7 (1,432/1,168)	1.07 (0.74–1.54)	<0.001/75.9	0.90 (0.33–2.43)	0.013/62.9	**1.40 (1.06–1.85)**	0.150/36.4	0.75 (0.29–1.95)	<0.001/75.4	**0.67 (0.54–0.82)**	0.005/60.5
Type of control											
Healthy	10 (2,149/1931)	1.25 (0.99–1.57)	0.001/69.4	1.31 (0.73–2.38)	0.010/58.5	**1.34 (1.07–1.68)**	0.039/49.1	1.16 (0.66–2.03)	0.002/65.5	0.82 (0.64–1.05)	0.006/60.7
Non-healthy	4 (230/298)	1.11 (0.32–3.83)	0.001/85.3	1.97 (0.10–37.66)	0.010/78.3	1.03 (0.58–1.83)	0.035/44.8	2.32 (0.13–40.73)	<0.001/71.5	0.82 (0.31–2.14)	0.095/56.7
Overall	9 (1700/1,628)	1.40 (1.16–1.68)	0.074/44.1	1.96 (1.20–3.21)	0.131/35.9	1.45 (1.22–1.72)	0.339/11.2	1.59 (0.92–2.74)	0.011/59.6	0.85 (0.64–1.12)	0.006/63.0
Ethnicity											
Caucasian	4 (538/523)	**1.38 (1.08–1.76)**	0.970/0.0	1.33 (0.49–3.58)	0.317/15.1	**1.35 (1.04–1.74)**	0.576/0.0	1.18 (0.43–3.28)	0.300/18.2	**0.68 (0.51–0.90)**	0.869/0.0
Asian	4 (1,012/1,055)	1.29 (0.90–1.83)	0.004/77.1	**2.00 (1.09–3.67)**	0.096/52.7	1.41 (0.98–2.04)	0.105/51.1	1.56 (0.78–3.10)	0.004/77.5	0.94 (0.56–1.58)	0.001/82.4
African	1 (150/50)	**1.83(1.05–3.19)**	NA	**9.14 (1.19–70.41)**	NA	1.40 (0.72–2.71)	NA	**9.33 (1.23–70.88)**	NA	1.32 (0.67–2.63)	NA
**Sex**											
Male	1 (51/72)	1.36 (0.71–2.63)	NA	1.59 (0.21–11.88)	NA	1.44 (0.67–3.09)	NA	1.43 (0.19–10.49)	NA	0.72 (0.33–1.57)	NA
Female	8 (1,649/1,556)	**1.39 (1.14–1.70)**	0.047/50.8	**1.96 (1.15–3.35)**	0.089/43.4	**1.42 (1.17–1.72)**	0.256/21.0	1.60 (0.89–2.88)	0.006/64.6	0.86 (0.64–1.16)	0.003/67.4
Female menopause											
NP	3 (551/479)	**1.65 (1.34–2.05)**	0.719/0.0	**2.62 (1.55–4.42)**	0.438/0.0	**1.35 (1.00–1.81)**	0.340/10.6	**2.23 (1.49–3.34)**	0.352/4.2	1.18 (0.70–1.98)	0.036/70.1
*p*	5 (1,098/1,077)	1.20 (0.86–1.67)	0.015/67.5	1.21 (0.43–3.41)	0.037/60.9	**1.43 (1.08–1.89)**	0.195/34.0	0.99 (0.34–2.89)	0.005/73.4	**0.70 (0.56–0.88)**	0.307/16.9
Type of control											
Healthy	8 (1,550/1,578)	**1.36 (1.11–1.66)**	0.055/49.2	1.83 (1.13–2.98)	0.157/34.0	**1.55 (1.33–1.82)**	0.501/0.0	1.44 (0.84–2.48)	0.016/59.4	0.81 (0.61–1.09)	0.006/64.4
Non-healthy	1 (150/50)	**1.83 (1.05–3.19)**	NA	**9.14 (1.19–70.41)**	NA	1.06 (0.61–1.81)	0.242/27.0	**9.33 (1.23–70.88)**	NA	1.32 (0.67–2.63)	NA
Egger’s test											
***P***_***E***_		0.217	0.191	0.187	0.382	0.880					

OPG A163G: allele model: G vs A, additive model: GG, vs; AA, dominant model: AG + GG, vs; AA, recessive model; GG, vs AA + AG, over-dominant model: AA + GG, vs AG; HWE, Hardy–Weinberg equilibrium; *OPG*, osteoprotegerin; *p* = Postmenopausal women; NP, Non-postmenopausal women. Bold values indicated that these results are statistically significant.

**FIGURE 3:**
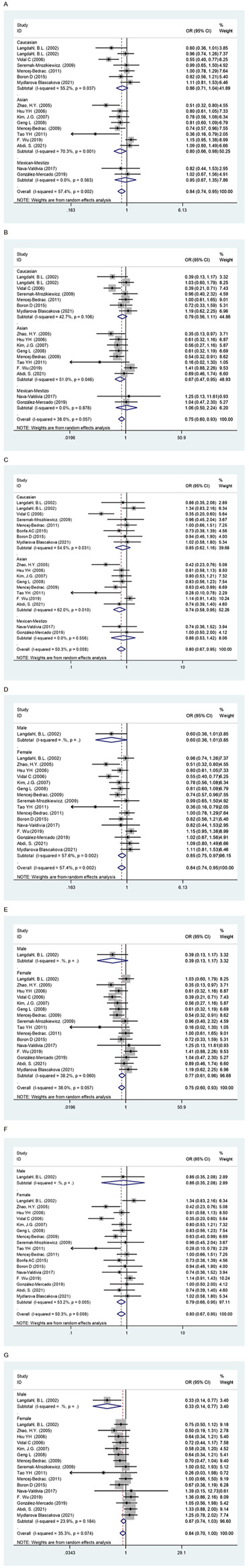
The forest plots of all selected studies on the adjusted association between the OPG G1181C polymorphism and risk of osteoporosis in overall and subgroup analyses by ethnicity and sex (A: C vs G; **(B)** CC vs GG; **(C)** GC + CC vs GG; **(D)** C vs G; **(E)** CC vs GG; **(F)** GC + CC vs GG; **(G)** CC vs GG + GC).

Overall, the *OPG* G1181C polymorphism reduces the risk of osteoporosis (C vs G: OR = 0.84, 95% CI = 0.74–0.95; CC vs GG: OR = 0.75, 95% CI = 0.60–0.93; ​GC + CC vs GG: OR = 0.80, 95% CI = 0.67–0.95; CC vs GG + GC: OR = 0.84, 95% CI = 0.70–1.00, Table 4 and Figure 3). In subgroup analyses, the risk of osteoporosis was also significantly lower in Asian (C vs G: OR = 0.80, 95% CI = 0.66–0.98; CC vs GG: OR = 0.67, 95% CI = 0.47–0.95; ​GC + CC vs GG: OR = 0.74, 95% CI = 0.58–0.95, Table 4 and Figure 3) and female (C vs G: OR = 0.85, 95% CI = 0.75–0.97; CC vs GG: OR = 0.77, 95% CI = 0.61–0.96; ​GC + CC vs GG: OR = 0.79, 95% CI = 0.66–0.95, Table 4 and Figure 3). On this basis, we further found that *OPG* G1181C polymorphism could reduce the risk of osteoporosis in premenopausal female (C vs G: OR = 0.86, 95% CI = 0.73–1.00; CC vs GG + GC: OR = 0.71, 95% CI = 0.52–0.95, Table 4 and Figure 3).

Finally, we found that the *OPG* T245 and T950C polymorphisms did not increase or decrease the prevalence of osteoporosis in both global and subgroup analyses, that is, there was no association between them (Tables 2 and 3).

**TABLE 2:** Meta-analysis of the association of OPG T245G polymorphism with risk of osteoporosis.

Variable	n (Cases/Controls)	G vs T		GG vs TT		TG + GG vs TT		GG vs TT + TG		TT + GG vs TG	
		OR (95%CI)	Ph/I2 (%)	OR (95%CI)	Ph/I2 (%)	OR (95%CI)	Ph/I2 (%)	OR (95%CI)	Ph/I2 (%)	OR (95%CI)	Ph/I2 (%)
Overall	9 (941/1,019)	1.17 (0.83–1.63)	0.176/33.0	0.66 (0.18–2.43)	0.195/36.2	1.10 (0.76–1.59)	0.166/32.8	0.66 (0.23–1.90)	0.145/41.5	0.72 (0.54–0.96)	0.738/0.0
Ethnicity											
Caucasian	6 (602/773)	1.16 (0.56–2.41)	0.044/62.9	0.71 (0.03–20.41)	0.050/73.9	1.05 (0.60–1.86)	0.054/57.0	0.43 (0.07–2.55)	0.148/47.6	0.60 (0.40–0.90)	0.813/0.0
Asian	2 (295/224)	1.15 (0.80–1.65)	0.400/0.0	0.73 (0.16–3.35)	NA	1.28 (0.77–2.10)	0.400/0.0	1.04 (0.52–2.06)	NA	0.86 (0.56–1.30)	0.323/0.0
Mexican-Mestizo	1 (44/22)	1.54 (0.30–7.94)	NA	1.56 (0.06–39.95)	NA	1.28 (0.23–7.20)	NA	1.55 (0.06–39.65)	NA	1.00 (0.17–5.93)	NA
Sex											
Male	1 (51/72)	0.93 (0.51–1.70)	NA	NA	NA	1.46 (0.40–5.32)	NA	NA	NA	0.69 (0.19–2.51)	NA
Female	7 (869/926)	1.19 (0.82–1.73)	0.114/46.3	0.66 (0.18–2.43)	0.195/36.2	1.08 (0.72–1.62)	0.114/41.5	0.78 (0.23–2.69)	0.113/49.7	0.73 (0.54–0.98)	0.583/0.0
Mix	1 (21/21)	0.28 (0.03–2.86)	NA	NA	NA	NA	NA	0.27 (0.03–2.81)	NA	0.27 (0.03–2.81)	NA
Female menopause											
NP	3 (311/399)	2.13 (1.16–3.89)	0.676/0.0	3.02 (0.33–27.77)	0.582/0.0	1.08 (0.35–3.32)	0.011/77.7	2.91 (0.32–26.76)	0.600/0.0	0.55 (0.29–1.04)	0.481/0.0
*p*	4 (558/527)	1.00 (0.74–1.35)	0.322/11.9	0.37 (0.09–1.44)	0.202/38.6	1.12 (0.79–1.59)	0.736/0.0	0.49 (0.09–2.78)	0.034/77.6	0.79 (0.56–1.11)	0.504/0.0
Type of control											
Healthy	6 (826/779)	1.16 (0.80–1.67)	0.117/43.3	0.61 (0.13–3.00)	0.119/53.1	1.32 (0.97–1.79)	0.425/0.0	0.61 (0.18–2.07)	0.083/55.0	0.71 (0.53–0.95)	0.638/0.0
Non-healthy	3 (115/240)	1.54 (0.30–7.94)	NA	1.56 (0.06–39.95)	NA	0.59 (0.32–1.09)	0.545/32.8	1.55 (0.06–39.65)	NA	1.00 (0.17–5.93)	NA
Sensitivity analysis											
HWE and Quality score >12											
Overall	5 (606/535)	1.36 (1.00–1.83)	0.427/0.0	1.15 (0.33–4.03)	0.500/0.0	1.52 (1.04–2.21)	0.685/0.0	1.14 (0.59–2.18)	0.595/0.0	0.74 (0.53–1.04)	0.593/0.0
Ethnicity											
Caucasian	2 (267/289)	2.04 (1.15–3.64)	0.539/0.0	5.40 (0.26–113.16)	NA	1.99 (1.09–3.61)	0.597/0.0	5.07 (0.24–106.22)	NA	0.54 (0.30–0.98)	0.679/0.0
Asian	2 (295/224)	1.15 (0.80–1.65)	0.400/0.0	0.73 (0.16–3.35)	NA	1.28 (0.77–2.10)	0.400/0.0	1.04 (0.52–2.06)	NA	0.86 (0.56–1.30)	0.323/0.0
Mexican-Mestizo	1 (44/22)	1.54 (0.39–7.94)	NA	1.56 (0.06–39.95)	NA	1.28 (0.23–7.20)	NA	1.55 (0.06–39.65)	NA	1.00 (0.17–5.93)	NA
Sex											
Male	1 (51/72)	1.43 (0.40–5.08)	NA	NA	NA	1.46 (0.40–5.32)	NA	NA	NA	0.69 (0.19–2.51)	NA
Female	4 (555/463)	1.37 (0.95–1.98)	0.280/21.8	1.15 (0.33–4.03)	0.500/0.0	1.52 (1.03–2.25)	0.518/0.0	1.14 (0.59–2.18)	0.595/0.0	0.75 (0.53–1.06)	0.427/0.0
Female menopause											
NP	2 (260/239)	2.13 (1.16–3.89)	0.676/0.0	3.02 (0.33–27.77)	0.582/0.0	2.02 (1.08–3.77)	0.581/0.0	2.91 (0.32–26.76)	0.600/0.0	0.55 (0.29–1.04)	0.481/0.0
*p*	2 (295/224)	1.15 (0.80–1.65)	0.400/0.0	0.73 (0.16–3.35)	NA	1.28 (0.77–2.10)	0.400/0.0	1.04 (0.52–2.06)	NA	0.86 (0.56–1.30)	0.323/0.0
Type of control											
Healthy	4 (562/513)	1.37 (0.96–1.96)	0.281/21.5	1.28 (0.22–7.53)	0.245/26.2	1.53 (1.04–2.24)	0.525/0.0	1.12 (0.57–2.20)	0.317/0.2	0.74 (0.52–1.04)	0.443/0.0
Non-healthy	1 (44/22)	1.54 (0.30–7.94)	NA	1.56 (0.06–39.95)	NA	1.28 (0.23–7.20)	NA	1.55 (0.06–39.65)	NA	1.00 (0.17–5.93)	NA
Egger’s test											
PE		0.928	0.247	0.757	0.822	0.705					

OPG, T245G: allele model: G vs T, additive model: GG, vs; TT, dominant model: TG + GG, vs; TT, recessive model; GG, vs TT + TG, over-dominant model: TT + GG, vs TG; HWE, Hardy–Weinberg equilibrium; OPG, Osteoprotegerin; *p* = Postmenopausal women; NP, Non-postmenopausal women.

**TABLE 3:** Meta-analysis of the association of *OPG* T950C polymorphism with risk of osteoporosis.

Variable	n (Cases/Controls)	C vs T		CC vs TT		TC + CC vs TT		CC vs TT + TC		TT + CC vs TC	
		OR (95%CI)	*Ph/I* ^*2*^ (%)	OR (95%CI)	*Ph/I* ^*2*^ (%)	OR (95%CI)	*Ph/I* ^*2*^ (%)	OR (95%CI)	*Ph/I* ^*2*^ (%)	OR (95%CI)	*Ph/I* ^*2*^ (%)
Overall	12 (1,610/1,234)	0.93 (0.71–1.23)	<0.001/82.4	0.91 (0.55–1.51)	<0.001/77.6	0.90 (0.63–1.28)	<0.001/73.1	0.96 (0.67–1.37)	<0.001/68.8	1.02 (0.88–1.19)	0.764/0.0
Ethnicity											
Caucasian	4 (753/467)	0.68 (0.37–1.24)	<0.001/91.0	0.52 (0.19–1.41)	<0.001//87.1	0.65 (0.27–1.53)	<0.001/87.8	0.61 (0.34–1.08)	0.007/75.1	0.92 (0.72–1.18)	0.485/0.0
Asian	7 (821/749)	1.14 (0.91–1.44)	<0.001/82.4	1.33 (0.82–2.17)	0.039/54.9	1.10 (0.80–1.51)	0.085/46.0	1.30 (0.93–1.83)	0.178/32.7	1.10 (0.90–1.34)	0.707/0.0
Mexican-Mestizo	1 (36/18)	0.78 (0.33–1.83)	NA	0.59 (0.10–3.57)	NA	0.63 (0.11–3.46)	NA	0.80 (0.26–2.49)	NA	1.00 (0.31–13.19)	NA
Sex											
Male	2 (149/173)	1.08 (0.49–2.38)	0.014/83.3	1.13 (0.25–5.15)	0.018/82.2	1.05 (0.54–2.04)	0.180/44.3	1.17 (0.29–4.67)	0.012/84.3	1.18 (0.75–1.87)	0.300/6.9
Female	8 (1,124/792)	0.83 (0.57–1.20)	<0.001/84.6	0.74 (0.38–1.44)	<0.001/78.9	0.78 (0.47–1.28)	<0.001/79.0	0.82 (0.53–1.26)	0.005/65.5	1.01 (0.83–1.22)	0.571/0.0
Mix	2 (272/208)	1.23 (0.72–2.09)	<0.001/72.9	1.59 (0.49–5.17)	0.047/74.7	1.31 (0.63–2.76)	0.092/64.7	1.29 (0.61–2.76)	0.123/58.0	0.98 (0.71–1.35)	0.768/0.0
Female menopause											
NP	4 (743/508)	0.97 (0.82–1.15)	0.957/0.0	0.97 (0.69–1.36)	0.958/0.0	0.98 (0.75–1.29)	0.675/0.0	0.94 (0.71–1.24)	0.791/0.0	0.97 (0.75–1.27)	0.331/12.4
*p*	4 (381/284)	0.74 (0.33–1.66)	<0.001/91.3	0.64 (0.15–2.71)	<0.001/85.5	0.64 (0.22–1.86)	<0.001/88.7	0.77 (0.30–1.99)	0.008/74.4	1.08 (0.79–1.48)	0.566/0.0
Type of control											
Healthy	9 (1,459/1,105)	0.93 (0.68–1.27)	<0.001/85.3	0.91 (0.52–1.59)	<0.001/80.4	0.90 (0.62–1.33)	<0.001/76.0	0.94 (0.62–1.42)	<0.001/74.3	1.01 (0.86–1.19)	0.606/0.0
Non-healthy	3 (151/129)	0.94 (0.46–1.92)	0.019/74.8	0.91 (0.20–4.13)	0.019/77.6	0.84 (0.25–2.81)	0.023/73.4	1.05 (0.48–2.30)	0.161/45.2	1.10 (0.68–1.77)	0.619/0.0
**Sensitivity analysis**											
**HWE and Quality score > 12**											
Overall	7 (807/762)	0.85 (0.55–1.32)	<0.001/87.5	0.79 (0.36–1.73)	<0.001/83.2	0.84 (0.47–1.49)	<0.001/82.0	0.82 (0.49–1.38)	0.001/73.4	0.98 (0.80–1.20)	0.568/0.0
Ethnicity											
Caucasian	3 (447/408)	0.59 (0.27–1.31)	<0.001/93.1	0.41 (0.11–1.51)	<0.001//89.9	0.55 (0.17–1.76)	<0.001/91.2	0.51 (0.25–1.03)	0.011/77.7	0.91 (0.67–1.24)	0.303/16.3
Asian	3 (324/336)	1.23 (0.86–1.75)	0.122/52.5	1.63 (0.72–3.66)	0.130/51.0	1.26 (0.72–2.19)	0.092/58.1	1.32 (0.88–2.00)	0.439/0.0	1.08 (0.79–1.46)	0.418/0.0
Mexican-Mestizo	1 (36/18)	0.78 (0.33–1.83)	NA	0.59 (0.10–3.57)	NA	0.63 (0.11–3.46)	NA	0.80 (0.26–2.49)	NA	1.00 (0.31–3.19)	NA
Sex											
Male	1 (51/72)	0.71 (0.43–1.18)	NA	0.50 (0.18–1.40)	NA	0.72 (0.33–1.57)	NA	0.56 (0.23–1.35)	NA	0.88 (0.43–1.81)	NA
Female	5 (691/629)	0.77 (0.45–1.32)	<0.001/89.7	0.66 (0.26–1.68)	<0.001/85.4	0.72 (0.35–1.48)	<0.001/85.6	0.75 (0.41–1.36)	0.002/76.3	1.00 (0.78–1.27)	0.327/13.6
Mix	1 (65/61)	**1.69 (1.03–2.79)**	NA	**3.23 (1.08–9.66)**	NA	2.10 (0.94–4.70)	NA	2.16 (0.85–5.50)	NA	0.89 (0.44–1.79)	NA
Female menopause											
NP	3 (437/449)	0.96 (0.80–1.16)	0.879/0.0	0.96 (0.66–1.40)	0.865/0.0	0.97 (0.72–1.31)	0.472/0.0	0.93 (0.68–1.26)	0.608/0.0	0.98 (0.66–1.44)	0.181/41.6
*p*	2 (254/180)	0.62 (0.14–2.70)	<0.001/95.5	0.50 (0.04–6.89)	0.001/90.5	0.53 (0.07–3.78)	<0.001/94.7	0.63 (0.11–3.47)	0.023/80.8	1.05 (0.70–1.60)	0.300/7.0
Type of control											
Healthy	5 (706/683)	0.76 (0.46–1.26)	<0.001/89.8	0.63 (0.27–1.52)	<0.001/85.5	0.73 (0.38–1.41)	<0.001/85.5	0.70 (0.39–1.25)	0.002/76.6	0.99 (0.78–1.25)	0.316/15.5
Non-healthy	2 (101/79)	1.24 (0.59–2.62)	0.125/57.4	1.62 (0.31–8.30)	0.114/60.0	1.46 (0.49–4.34)	0.209/36.7	1.39 (0.53–3.66)	0.184/43.3	0.92 (0.50–1.67)	0.865/0.0
**Egger’s test**											
***P***_***E***_		0.828		0.687		0.885		0.414		0.882	

OPG T950C: allele model: C vs T, additive model: CC, vs; TT, dominant model: TC + CC, vs; TT, recessive model; CC, vs TT + TC, over-dominant model: TT + CC, vs TC; HWE, Hardy–Weinberg equilibrium; *OPG*, osteoprotegerin; *p* = Postmenopausal women; NP, Non-postmenopausal women. Bold values indicated that these results are statistically significant.

### Heterogeneity and sensitivity analyses

​During the statistical process, we found several possible sources of heterogeneity, including ethnicity, sex, sample size, female menopause, control type, quality score and HWE. Therefore, we used meta-regression analysis to determine the causes of heterogeneity. A meta-regression analysis revealed that the sample size (G vs A: *p* = 0.023) and HWE (G vs A: *p* = 0.010) were the source of heterogeneity between the *OPG* A163G polymorphism and the risk of osteoporosis. The type of controls (TG + GG vs. TT: *p* = 0.006) was the sources of heterogeneity between the *OPG* T245G polymorphism and the risk of osteoporosis. At the same time, the female menopause (C vs T: *p* = 0.016; CC vs. TT: *p* = 0.041; TC + CC vs. TT: *p* = 0.020; CC vs. TT + TC: *p* = 0.038), sample size (TC + CC vs. TT: *p* = 0.037) and sex (TT + CC vs. TC: *p* = 0.045) were the sources of heterogeneity between the *OPG* T950C polymorphism and the risk of osteoporosis. For the *OPG* G1181C, no covariate was found as a possible cause of between-study variation.

In this meta-analysis, two methods were used for sensitivity analysis. In the first, each study was removed one by one, and the results did not change when a single study was removed each time. Second, literature that met both high quality scores and HWE was screened. When only control studies that met HWE and high-quality scores were retained, we found that in the overall analysis, the *OPG* A163G polymorphism significantly increases the risk of osteoporosis (G vs A: OR = 1.40, 95% CI = 1.16–1.68; GG vs AA: OR = 1.96, 95% CI = 1.20–3.21; AG + GG vs AA: OR = 1.45, 95% CI = 1.22–1.72, Table 1), and the same was observed in the female population (G vs A: OR = 1.39, 95% CI = 1.14–1.70; GG vs AA: OR = 1.96, 95% CI = 1.15–3.35; AG + GG vs AA: OR = 1.42, 95% CI = 1.17–1.72, Table 1). However, we did not find significant changes in *OPG* T245G, T950C and G1181C.

### Publication bias

**FIGURE 4:**
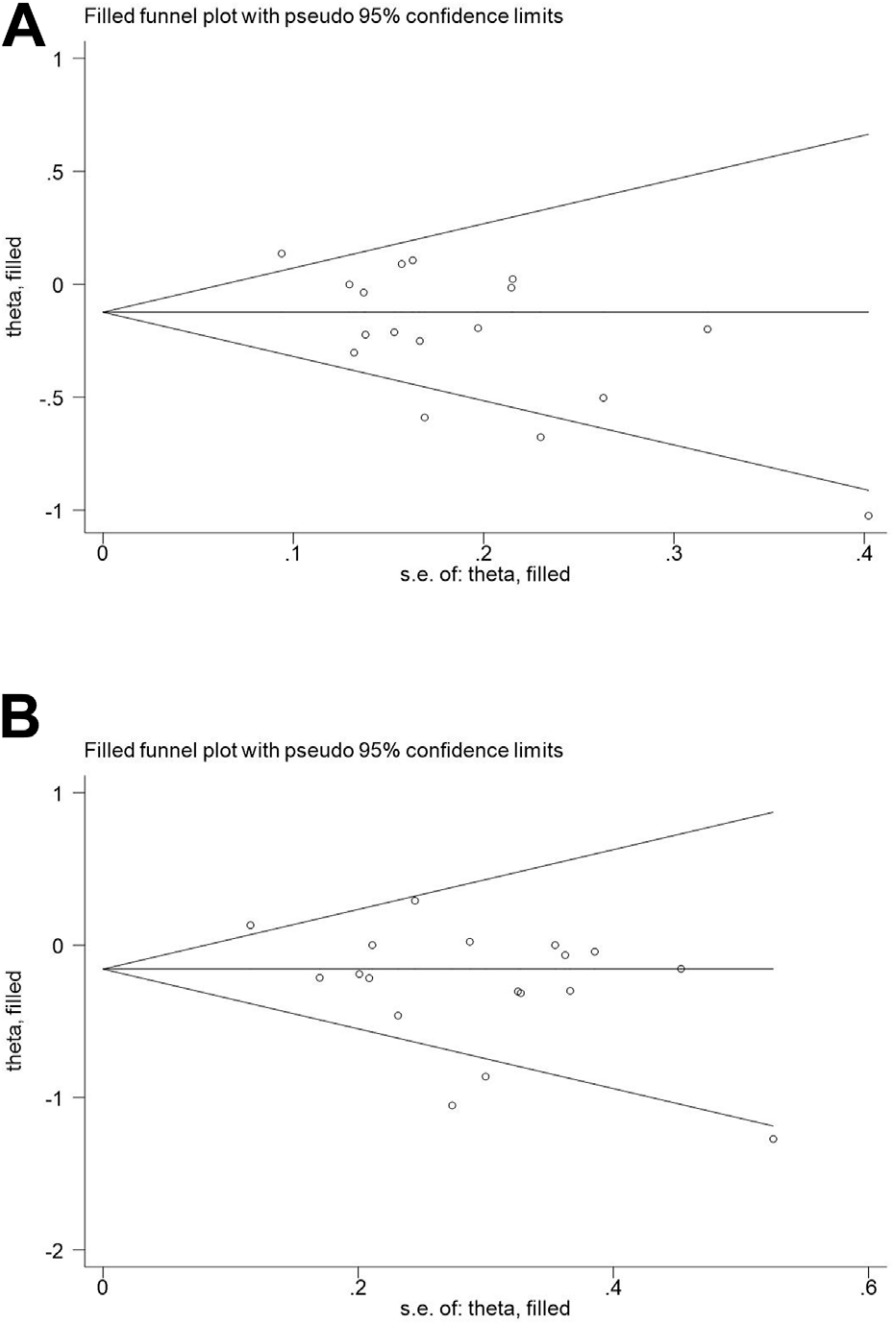
Begg’s funnel plot to assess publication bias **(A)**: C vs G; **(B)** GC + CC vs. GG.

The results of Begg’s funnel plot and Egger’s test showed that only *OPG* G1181C polymorphism and risk of osteoporosis had publication bias (C vs G: *p* = 0.015; GC + CC vs. GG: *p* = 0.045, Table 4). Then, publication bias was adjusted using the nonparametric “trim and fill”method. However, when we applied a nonparametric “trim and fill” method (Figure 4), the analysis showed that no more study should be added, showing that the results were stable.

**TABLE 4:** Meta-analysis of the association of *OPG* G1181C polymorphism with risk of osteoporosis.

Variable	n (Cases/Controls)	C vs G		CC vs GG		GC + CC vs GG		CC vs GG + GC		GG + CC vs GC	
		OR (95%CI)	*Ph/I* ^*2*^ (%)	OR (95%CI)	*Ph/I* ^*2*^ (%)	OR (95%CI)	*Ph/I* ^*2*^ (%)	OR (95%CI)	*Ph/I* ^*2*^ (%)	OR (95%CI)	*Ph/I* ^*2*^ (%)
Overall	18 (3,472/3,035)	**0.84 (0.74–0.95)**	0.002/57.4	**0.75 (0.60–0.93)**	0.057/38.0	**0.80 (0.67–0.95)**	0.008/50.3	**0.84 (0.70–1.00)**	0.074/35.3	1.08 (0.92–1.25)	0.013/48.7
Ethnicity											
Caucasian	8 (1,333/1,103)	0.86 (0.71–1.04)	0.037/55.2	0.79 (0.56–1.11)	0.106/42.7	0.85 (0.62–1.16)	0.031/54.5	0.82 (0.64–1.05)	0.144/37.4	0.93 (0.68–1.27)	0.007/66.0
Asian	8 (1921/1800)	**0.80 (0.66–0.98)**	0.001/70.3	**0.67 (0.47–0.95)**	0.046/51.0	**0.74 (0.58–0.95)**	0.010/62.0	0.81 (0.59–1.10)	0.043/51.6	1.15 (0.96–1.37)	0.143/35.8
Mexican-Mestizo	2 (218/132)	0.95 (0.67–1.35)	0.563/0.0	1.06 (0.50–2.24)	0.878/0.0	0.86 (0.53–1.42)	0.556/0.0	1.08 (0.59–1.98)	0.816/0.0	1.19 (0.75–1.89)	0.523/0.0
Sex											
Male	1 (50/72)	0.60 (0.36–1.01)	NA	0.39 (0.13–1.17)	NA	0.86 (0.35–2.08)	NA	**0.33 (0.14–0.77)**	NA	**0.45 (0.22–0.94)**	NA
Female	17 (3,422/2,963)	**0.85 (0.75–0.97)**	0.002/57.6	**0.77 (0.61–0.96)**	0.060/38.2	**0.79 (0.66–0.95)**	0.005/53.2	0.87 (0.74–1.03)	0.184/23.9	1.10 (0.95–1.27)	0.038/42.3
Female menopause											
NP	5 (869/926)	**0.86 (0.73–1.00)**	0.764/0.0	0.77 (0.54–1.09)	0.520/0.0	0.87 (0.71–1.07)	0.428/0.0	**0.71 (0.52–0.95)**	0.899/0.0	0.96 (0.71–1.30)	0.114/49.5
*p*	12 (2,553/2037)	0.85 (0.72–1.00)	0.002/67.5	0.76 (0.57–1.01)	0.024/50.0	**0.75 (0.58–0.96)**	0.001/63.7	0.92 (0.76–1.12)	0.129/32.7	1.16 (0.98–1.37)	0.079/39.3
Type of control											
Healthy	16 (3,290/2,830)	**0.84 (0.74–0.96)**	0.001/60.0	**0.74 (0.59–0.93)**	0.042/41.5	**0.80 (0.66–0.96)**	0.004/55.6	**0.83 (0.70–1.00)**	0.056/38.9	1.07 (0.91–1.25)	0.010/50.8
Non-healthy	2 (182/205)	0.82 (0.44–1.53)	NA	1.25 (0.13–11.61)	NA	0.73 (0.46–1.18)	0.975/0.0	1.39 (0.15–12.73)	NA	1.43 (0.69–2.97)	NA
**Sensitivity analysis**											
**HWE and Quality score > 12**											
Overall	15 (3,036/2,791)	**0.86 (0.76–0.98)**	0.004/56.6	**0.76 (0.61–0.96)**	0.052/40.6	**0.82 (0.68–0.98)**	0.010/52.2	0.86 (0.71–1.03)	0.067/38.0	1.07 (0.92–1.25)	0.025/46.5
Ethnicity											
Caucasian	6 (968/879)	0.86 (0.69–1.08)	0.021/62.2	0.79 (0.54–1.17)	0.065/51.8	0.86 (0.57–1.29)	0.010/66.8	0.84 (0.63–1.11)	0.111/44.2	0.96 (0.68–1.38)	0.005/70.2
Asian	7 (1850/1780)	0.84 (0.70–1.02)	0.005/67.7	**0.70 (0.49–0.99)**	0.056/51.0	**0.79 (0.62–0.99)**	0.031/56.8	0.83 (0.61–1.13)	0.042/54.2	1.11 (0.94–1.30)	0.253/23.1
Mexican-Mestizo	2 (218/132)	0.95 (0.67–1.35)	0.563/0.0	1.06 (0.50–2.24)	0.878/0.0	0.86 (0.53–1.42)	0.556/0.0	1.08 (0.59–1.98)	0.816/0.0	1.19 (0.75–1.89)	0.523/0.0
Sex											
Male	1 (50/72)	0.60 (0.36–1.01)	NA	0.39 (0.13–1.17)	NA	0.86 (0.35–2.08)	NA	**0.33 (0.14–0.77)**	NA	**0.45 (0.22–0.94)**	NA
Female	14 (2,986/2,719)	**0.87 (0.77–0.99)**	0.005/56.6	**0,78 (0.62–0.99)**	0.056/40.8	**0.81 (0.67–0.98)**	0.006/55.6	0.90 (0.76–1.06)	0.185/24.9	1.10 (0.95–1.27)	0.074/37.9
Female menopause											
NP	4 (818/766)	**0.86 (0.73–1.00)**	0.764/0.0	0.77 (0.54–1.09)	0.520/0.0	0.90 (0.71–1.14)	0.321/14.2	**0.71 (0.52–0.95)**	0.899/0.0	0.96 (0.71–1.30)	0.114/49.5
*p*	10 (2,168/1953)	0.88 (0.74–1.04)	0.001/68.1	0.78 (0.58–1.06)	0.020/54.2	**0.77 (0.59–0.99)**	0.002/65.0	0.96 (0.79–1.17)	0.149/32.4	1.16 (0.99–1.36)	0.168/30.2
Type of control											
Healthy	14 (2,905/2,746)	**0.86 (0.76–0.98)**	0.002/59.6	**0.76 (0.60–0.96)**	0.037/44.5	**0.82 (0.68–0.99)**	0.006/55.3	0.85 (0.71–1.03)	0.049/42.0	1.06 (0.91–1.25)	0.020/48.9
Non-healthy	1 (131/45)	0.82 (0.44–1.53)	NA	1.25 (0.13–11.61)	NA	0.74 (0.36–1.52)	NA	1.39 (0.15–12.73)	NA	1.43 (0.69–2.97)	NA
**Egger’s test**											
***P***_***E***_		**0.015**	0.124	**0.045**	0.094	0.271					

OPG G1181C: allele model: C vs G, additive model: CC, vs; GG, dominant model: GC + CC, vs; GG, recessive model; CC, vs GG + GC, over-dominant model: GG + CC, vs GC; HWE, Hardy–Weinberg equilibrium; *OPG*, osteoprotegerin; *p* = Postmenopausal women; NP, Non-postmenopausal women. Bold values indicated that these results are statistically significant.

### Credibility of previous meta-analyses

Since the previously published meta-analyses were not evaluated for credibility, we evaluated the results for accuracy, applying the FPRP, BFDP, and Venice criteria. Through calculation and statistics, we obtained results that Guo et al., 2014, Luo et al., 2014, Li et al., 2017 and Li et al., 2021 were classified as less credible. Table 5 specifically shows the previously published credibility results (FPRP >0.2 and BFDP >0.8, I2 > 50%, statistical power <80%).

**TABLE 5:** Credibility of previously published meta-analysis.

Author	Gene	Model	N	Case/control	Variable	OR (95%CI)	Ph/*I* ^*2*^ (%)	Credibility		
								Prior probability of 0.001		
								Power	FPRP	BFDP
Guo et al. (2014)	*OPG* A163G	G vs A	7	1,078/1,092	Overall	1.25 (1.07–1.45)	<0.001/77.2	0.535	0.999	0.991
Guo et al. (2014)	*OPG* A163G	AG + GG vs AA	7	1,078/1,092	Overall	1.28 (1.06–1.55)	0.062/50.0	0.948	0.924	0.996
Guo et al. (2014)	*OPG* A163G	GG vs AA	7	1,078/1,092	Overall	1.50 (1.06–2.11)	0.003/70.3	0.500	0.975	0.000
Guo et al. (2014)	*OPG* A163G	G vs A	3	416/383	Caucasian	1.45 (1.10–1.92)	NA	0.594	0.941	0.994
Guo et al. (2014)	*OPG* A163G	AG + GG vs AA	3	416/383	Caucasian	1.47 (1.07–2.01)	NA	0.550	0.966	0.996
Guo et al. (2014)	*OPG* G1181C	C vs C	6	1,287/1,105	Overall	0.79 (0.70–0.89)	0.334/12.6	0.997	0.096	0.847
Guo et al. (2014)	*OPG* G1181C	GC + CC vs GG	6	1,287/1,105	Overall	0.79 (0.66–0.95)	0.087/48.1	0.964	0.927	0.997
Guo et al. (2014)	*OPG* G1181C	CC vs GG + GC	6	1,287/1,105	Overall	0.73 (0.59–0.91)	0.513/0.0	0.790	0.866	0.992
Guo et al. (2014)	*OPG* G1181C	CC vs GG	6	1,287/1,105	Overall	0.65 (0.50–0.85)	0.539/0.0	0.427	0.794	0.975
Guo et al. (2014)	*OPG* G1181C	CC vs GC	6	1,287/1,105	Overall	0.70 (0.56–0.88)	0.748/0.0	0.662	0.773	0.982
Guo et al. (2014)	*OPG* G1181C	C vs C	3	646/581	Caucasian	0.83 (0.71–0.98)	NA	0.995	0.966	0.998
Guo et al. (2014)	*OPG* G1181C	CC vs GC	3	646/581	Caucasian	0.70 (0.54–0.91)	NA	0.642	0.923	0.993
Guo et al. (2014)	*OPG* G1181C	C vs C	4	641/524	Asians	0.73 (0.61–0.89)	NA	0.815	0.694	0.981
Guo et al. (2014)	*OPG* G1181C	GC + CC vs GG	4	641/524	Asians	0.73 (0.57–0.92)	NA	0.779	0.908	0.994
Guo et al. (2014)	*OPG* G1181C	CC vs GG + GC	4	641/524	Asians	0.59 (0.39–0.91)	NA	0.290	0.983	0.996
Guo et al. (2014)	*OPG* G1181C	CC vs GG	4	641/524	Asians	0.54 (0.35–0.83)	NA	0.168	0.967	0.989
Luo et al. (2014)	*OPG* A163G	G vs A	7	1,078/1,092	Overall	1.25 (1.07–1.45)	NA	0.535	0.999	0.991
Luo et al. (2014)	*OPG* A163G	AG + GG vs AA	7	1,078/1,092	Overall	1.28 (1.06–1.55)	NA	0.948	0.924	0.996
Luo et al. (2014)	*OPG* A163G	GG vs AA	7	1,078/1,092	Overall	1.50 (1.06–2.11)	NA	0.500	0.975	0.000
Luo et al. (2014)	*OPG* A163G	G vs A	3	416/383	Caucasian	1.45 (1.10–1.92)	NA	0.594	0.941	0.994
Luo et al. (2014)	*OPG* A163G	AG + GG vs AA	3	416/383	Caucasian	1.47 (1.07–2.01)	NA	0.550	0.966	0.996
Luo et al. (2014)	*OPG* G1181C	C vs C	5	1,002/815	Overall	0.79 (0.69–0.90)	NA	0.995	0.284	0.945
Luo et al. (2014)	*OPG* G1181C	GC + CC vs GG	5	1,002/815	Overall	0.79 (0.64–0.98)	NA	0.939	0.972	0.998
Luo et al. (2014)	*OPG* G1181C	CC vs GG + GC	5	1,002/815	Overall	0.74 (0.59–0.93)	NA	0.815	0.923	0.995
Luo et al. (2014)	*OPG* G1181C	CC vs GG	5	1,002/815	Overall	0.66 (0.50–0.88)	NA	0.473	0.907	0.989
Luo et al. (2014)	*OPG* G1181C	CC vs GC	5	1,002/815	Overall	0.70 (0.55–0.89)	NA	0.655	0.846	0.988
Luo et al. (2014)	*OPG* G1181C	C vs C	3	646/581	Caucasian	0.83 (0.71–0.98)	NA	0.995	0.966	0.998
Luo et al. (2014)	*OPG* G1181C	CC vs GC	3	646/581	Caucasian	0.70 (0.55–0.89)	NA	0.655	0.846	0.988
Luo et al. (2014)	*OPG* G1181C	C vs C	2	356/234	Asians	0.67 (0.52–0.88)	NA	0.514	0.886	0.988
Luo et al. (2014)	*OPG* G1181C	GC + CC vs GG	2	356/234	Asians	0.65 (0.47–0.91)	NA	0.441	0.965	0.995
Luo et al. (2014)	*OPG* G1181C	CC vs GG + GC	2	356/234	Asians	0.55 (0.31–0.98)	NA	0.257	0.994	0.998
Luo et al. (2014)	*OPG* G1181C	CC vs GG	2	356/234	Asians	0.48 (0.26–0.87)	NA	0.139	0.991	0.995
Li et al. (2021)	*OPG* A163G	G vs A	10	NA	Overall	1.45 (1.29–1.64)	NA/36.6	0.705	0.000	0.000
Li et al. (2021)	*OPG* A163G	GG + GA vs AA	12	NA	Overall	1.48 (1.29–1.70)	NA/0.0	0.575	0.000	0.002
Li et al. (2021)	*OPG* A163G	G vs A	7	NA	Caucasian	1.36 (1.14–1.63)	NA/0.0	0.856	0.505	0.966
Li et al. (2021)	*OPG* A163G	GG + GA vs AA	8	NA	Caucasian	1.34 (1.10–1.64)	NA/0.0	0.863	0.840	0.991
Li et al. (2021)	*OPG* A163G	GG vs GA + AA	7	NA	Caucasian	1.88 (1.09–3.23)	NA/30.8	0.207	0.991	0.996
Li et al. (2021)	*OPG* T245G	G vs T	7	NA	Overall	1.47 (1.16–1.86)	NA/9.3	0.567	0.701	0.972
Li et al. (2021)	*OPG* T245G	GG + GT vs TT	8	NA	Overall	1.65 (1.27–2.13)	NA/0.0	0.232	0.343	0.800
Li et al. (2021)	*OPG* T245G	G vs T	4	NA	Caucasian	1.60 (1.11–2.03)	NA/0.0	0.298	0.268	0.787
Li et al. (2021)	*OPG* T245G	GG + GT vs TT	5	NA	Caucasian	1.68 (1.20–2.35)	NA/0.0	0.254	0.906	0.981
Li et al. (2021)	*OPG* G1181C	CC + CG vs GG	13	NA	Overall	0.83 (0.68–1.00)	NA/43.5	0.989	0.981	0.999
Li et al. (2021)	*OPG* G1181C	CC + CG vs GG	13	NA	Caucasian	0.78 (0.64–0.94)	NA/0.0	0.950	0.905	0.995

HWE, Hardy–Weinberg equilibrium; *OPG*, osteoprotegerin; *p* = postmenopausal women; NP, Non-postmenopausal women; FPRP, false-positive report probabilities; BFDP, bayesian false discovery probability; NA: = not available.

### Credibility of the current meta-analysis

Our study also used BFDP, FPRP and Venice criteria to evaluate the statistically significant credibility. Associations that met the following criteria were considered to have high confidence: 1) at least two genetic models were statistically significant; 2) statistical power >80%; 3) *I* ^2^ < 50%; 4) FPRP <0.2 and BFDP <0.8. If all four criteria are not met, then all meaningful results are considered “less credible”. Table 6 specifically shows the results of the credibility assessment for all meaningful results. In the final calculation, we can find that all statistically significant results are “less likely".

**TABLE 6:** Credibility of the current meta-analysis.

Variables	Model	OR (95%CI)	I2 (%)	Statistical power	Credibility	
					Prior probability of 0.001	
					FPRP	BFDP
A163G						
Overall	AG + GG vs AA	1.29 (1.04–1.59)	44.8	0.921	0.949	0.997
Ethnicity						
Caucasian	AG + GG vs AA	1.35 (1.06–1.73)	3.6	0.797	0.957	0.997
	AA + GG vs AG	0.64 (0.49–0.82)	0.0	0.373	0.527	0.924
African	G vs A	1.83 (1.05–3.19)	NA	0.242	0.993	0.997
	GG vs AA	9.14 (1.19–70.41)	NA	0.041	0.999	0.999
	GG vs AA + AG	9.33 (1.23–70.88)	NA	0.039	0.999	0.999
Sex						
Female	G vs A	1.30 (1.03–1.64)	64.8	0.886	0.968	0.998
	AG + GG vs AA	1.42 (1.18–1.71)	18.8	0.718	0.232	0.886
Female menopause						
NP	G vs A	1.68 (1.36–2.07)	0.0	0.144	0.008	0.054
	GG vs AA	2.74 (1.64–4.58)	0.0	0.011	0.918	0.845
	AG + GG vs AA	1.37 (1.04–1.79)	0.0	0.747	0.966	0.997
	GG vs AA + AG	2.31 (1.55–3.43)	3.0	0.016	0.672	0.589
*p*	AG + GG vs AA	1.40 (1.06–1.85)	36.4	0.686	0.963	0.997
	AA + GG vs AG	0.67 (0.54–0.82)	60.5	0.519	0.164	0.788
Type of control						
Healthy	AG + GG vs AA	1.34 (1.07–1.68)	49.1	0.836	0.930	0.996
Sensitivity analysis						
HWE and Quality score >12						
Overall	G vs A	1.40 (1.16–1.68)	44.1	0.771	0.279	0.913
	GG vs AA	1.96 (1.20–3.21)	35.9	0.144	0.981	0.992
	AG + GG vs AA	1.45 (1.22–1.72)	11.2	0.651	0.030	0.475
Ethnicity						
Caucasian	G vs A	1.38 (1.08–1.76)	0.0	0.749	0.926	0.995
	AG + GG vs AA	1.35 (1.04–1.74)	0.0	0.792	0.963	0.997
	AA + GG vs AG	0.68 (0.51–0.90)	0.0	0.555	0.926	0.993
Asian	GG vs AA	2.00 (1.09–3.67)	52.7	0.176	0.993	0.997
African	G vs A	1.83 (1.05–3.19)	NA	0.242	0.993	0.997
	GG vs AA	9.14 (1.19–70.41)	NA	0.041	0.999	0.999
	GG vs AA + AG	9.33 (1.23–70.88)	NA	0.039	0.999	0.999
Sex						
Female	G vs A	1.39 (1.14–1.70)	50.8	0.771	0.636	0.975
	GG vs AA	1.96 (1.15–3.35)	43.4	0.164	0.988	0.995
	AG + GG vs AA	1.42 (1.17–1.72)	21.0	0.712	0.320	0.919
Female menopause						
NP	G vs A	1.65 (1.34–2.05)	0.0	0.195	0.030	0.216
	GG vs AA	2.62 (1.55–4.42)	0.0	0.018	0.944	0.920
	AG + GG vs AA	1.35 (1.00–1.81)	10.6	0.759	0.983	0.998
	GG vs AA + AG	2.23 (1.49–3.34)	4.2	0.027	0.786	0.786
*p*	AG + GG vs AA	1.43 (1.08–1.89)	34.0	0.632	0.950	0.995
	AA + GG vs AG	0.70 (0.56–0.88))	16.9	0.662	0.773	0.982
Type of control						
Healthy	G vs A	1.36 (1.11–1.66)	49.2	0.832	0.750	0.985
	AG + GG vs AA	1.55 (1.33–1.82)	0.0	0.344	<0.001	0.006
Non-healthy	G vs A	1.83 (1.05–3.19)	NA	0.242	0.993	0.997
	GG vs AA	9.14 (1.19–70.41)	NA	0.041	0.999	0.999
	GG vs AA + AG	9.33 (1.23–70.88)	NA	0.039	0.999	0.999
T245G						
Overall	TT + GG vs TG	0.72 (0.54–0.96)	0.0	0.700	0.973	0.997
Ethnicity						
Caucasian	TT + GG vs TG	0.60 (0.40–0.90)	0.0	0.305	0.978	0.995
Sex						
Female	TT + GG vs TG	0.73 (0.54–0.98)	0.0	0.727	0.980	0.998
Female menopause						
NP	G vs T	2.13 (1.16–3.89)	0.0	0.127	0.991	0.995
Type of control						
Healthy	TT + GG vs TG	0.71 (0.53–0.95)	0.0	0.664	0.970	0.997
Sensitivity analysis						
HWE and Quality score >12						
Overall	G vs T	1.36 (1.00–1.83)	0.0	0.706	0.964	0.998
	TG + GG vs TT	1.52 (1.04–2.21)	0.0	0.472	0.984	0.997
Ethnicity						
Caucasian	G vs T	2.04 (1.15–3.64)	0.0	0.149	0.991	0.995
	TG + GG vs TT	1.99 (1.09–3.61)	0.0	0.176	0.993	0.997
	TT + GG vs TG	0.54 (0.30–0.98)	0.0	0.244	0.994	0.998
Sex						
Female	TG + GG vs TT	1.52 (1.03–2.25)	0.0	0.474	0.987	0.998
Female’s type						
NP	G vs T	2.13 (1.16–3.89)	0.0	0.127	0.991	0.995
	TG + GG vs TT	2.02 (1.08–3.77)	0.0	0.175	0.994	0.997
T950C						
Sensitivity analysis						
HWE and Quality score >12						
Ethnicity						
Caucasian	CC vs TT + TC	0.55 (0.31–0.99)	68.2	0.261	0.994	0.998
Sex						
Mix	C vs T	1.69 (1.03–2.79)	NA	0.321	0.992	0.998
	CC vs TT	3.23 (1.08–9.66)	NA	0.085	0.998	0.998
G1181C						
Overall	C vs G	0.84 (0.74–0.95)	57.4	1.000	0.846	0.995
	CC vs GG	0.75 (0.60–0.93)	38.0	0.858	0.911	0.995
	GC + CC vs GG	0.80 (0.67–0.95)	50.3	0.981	0.918	0.996
	CC vs GG + GC	0.84 (0.70–1.00)	35.3	0.995	0.980	0.999
Ethnicity						
Asian	C vs G	0.80 (0.66–0.98)	70.3	0.961	0.970	0.998
	CC vs GG	0.67 (0.47–0.95)	51.0	0.511	0.980	0.997
	GC + CC vs GG	0.74 (0.58–0.95)	62.0	0.794	0.958	0.997
Sex						
Male	CC vs GG + GC	0.33 (0.14–0.77)	NA	0.052	0.995	0.995
	GG + CC vs GC	0.45 (0.22–0.94)	NA	0.148	0.996	0.997
Female	C vs G	0.85 (0.75–0.97)	57.6	1.000	0.941	0.998
	CC vs GG	0.77 (0.61–0.96)	38.2	0.900	0.957	0.997
	GC + CC vs GG	0.79 (0.66–0.95)	53.2	0.964	0.927	0.997
Female menopause						
NP	C vs G	0.86 (0.73–1.00)	0.0	1.000	0.980	0.999
	CC vs GG + GC	0.71 (0.52–0.95)	0.0	0.664	0.970	0.997
*p*	GC + CC vs GG	0.75 (0.58–0.96)	63.7	0.825	0.964	0.997
Type of control						
Healthy	C vs G	0.84 (0.74–0.96)	60.0	1.000	0.913	0.997
	CC vs GG	0.74 (0.59–0.93)	41.5	0.815	0.923	0.995
	GC + CC vs GG	0.80 (0.66–0.96)	55.6	0.975	0.944	0.997
	CC vs GG + GC	0.83 (0.70–1.00)	38.9	0.989	0.981	0.999
Sensitivity analysis						
HWE and Quality score >12						
Overall	C vs G	0.86 (0.76–0.98)	56.6	1.000	0.959	0.999
	CC vs GG	0.76 (0.61–0.96)	40.6	0.864	0.961	0.997
	GC + CC vs GG	0.82 (0.68–0.98)	52.2	0.989	0.967	0.998
Ethnicity						
Asian	CC vs GG	0.70 (0.49–0.99)	51.0	0.609	0.986	0.998
	GC + CC vs GG	0.79 (0.62–0.99)	56.8	0.930	0.978	0.998
Sex						
Male	CC vs GG + GC	0.33 (0.14–0.77)	NA	0.052	0.995	0.995
	GG + CC vs GC	0.45 (0.22–0.94)	NA	0.148	0.996	0.997
Female	C vs G	0.87 (0.77–0.99)	56.6	1.000	0.972	0.999
	CC vs GG	0.78 (0.62–0.99)	40.8	0.902	0.979	0.998
	GC + CC vs GG	0.81 (0.67–0.98)	55.6	0.977	0.969	0.998
Female menopause						
NP	C vs G	0.86 (0.73–1.00)	0.0	1.000	0.980	0.999
	CC vs GG + GC	0.71 (0.52–0.95)	0.0	0.664	0.970	0.997
*p*	GC + CC vs GG	0.77 (0.59–0.99)	65.0	0.869	0.979	0.998
Type of control						
Healthy	C vs G	0.86 (0.76–0.98)	59.6	1.000	0.959	0.999
	CC vs GG	0.76 (0.60–0.96)	44.5	0.864	0.961	0.997
	GC + CC vs GG	0.82 (0.68–0.99)	55.3	0.984	0.975	0.999

HWE, Hardy–Weinberg equilibrium; OPG, osteoprotegerin; *p* = postmenopausal women; NP, Non-postmenopausal women; FPRP, false-positive report probability; BFDP, bayesian false discovery probability.

## Discussion

Osteoporosis is a systemic skeletal disease associated with the action of multiple genes. Evidence from many studies indicates that *OPG* polymorphism has been considered one of the potential genetic factors for osteoporosis. The *OPG*/*RANKL*/*RANK* is the main signal transduction pathway in osteoporosis, which can regulate the differentiation, induction, activation and maintenance of osteoclasts (Zhao et al., 2020; Udagawa et al., 2021). Osteoclasts are formed by the combination of *RANKL* and *RANK* on the surface of osteoclasts to form a complex (Nakashima et al., 2011; McCabe et al., 2015). *OPG* prevents osteoclast formation by blocking *RANKL* binding site and preventing *RANKL* from forming complex with *RANK* receptor, thus inhibiting bone resorption and thereby avoiding the occurrence of osteoporosis (Kurinami et al., 2016; Maria et al., 2018). Previous studies have focused on four *OPG* polymorphisms: G1181C, located in the first exon, and A163G, T245G, and T950C, located in the promoter. (Langdahl et al., 2002). Many investigators have attempted to demonstrate specific potential relationships between *OPG* polymorphisms and osteoporosis. However, so far there has not been enough meaningful evidence to confirm a relationship, which may be due to various factors, for instance, the small sample size, ethnic and geographical differences and so on. Our meta-analysis is a valid way to avoid some shortcomings.

Overall, we did not find a significant association between *OPG* T245G and T950C polymorphisms and osteoporosis. While the *OPG* A163G and G1181 C polymorphisms were associated with the risk of osteoporosis in several subgroups. Moreover, when we retained only high-quality score literature and HWE, we found that only the *OPG* A163G polymorphism was associated with increased the risk of osteoporosis in overall and several subgroup analyses. However, the pooled *p*-value must be adjusted on the meta-analyses of the gene polymorphism with risk of disease because they applied several subgroups and genetic models at the expense of multiple comparisons (Attia et al., 2003). Therefore, we investigated the false positive results based on the FPRP, BFDP, and Venice criterion. FPRP is a method that uses multiple hypothesis testing to assess the likelihood of important outcomes in molecular epidemiological investigations. In 2007, Wakefield introduced a test called Bayesian error, which is more accurate in genetic epidemiological surveys. Many reasons can lead to the bias of the results, among which statistical power is an important factor, of which statistical power is an important factor. Many studies have demonstrated that when statistical power> 80%, higher statistical significance or lower false discovery rates can be achieved. However, when we used the above methods to assess the credibility of the current study, all statistically significant demerits were considered “not credible” (FPRP >0.2 and BFDP >0.8, *I* ^2^ > 50%, statistical power <80%), in other words, all statistically significant results were considered false-positive results.

Significant publication bias was only observed between the *OPG* G1181C polymorphism and risk of osteoporosis. As far as we know, some low quality and small sample sizes studies are common bias and errors. These studies are not always strict and the quality is poor, so the statistically significant small sample study is more likely to be accepted, it will be very easy to produce false positive results, making the conclusions of these original studies implausible. In the test of publication bias, the asymmetry of the funnel plot is caused by the study of low quality and small samples.

All four previous meta-analyses researched the correlation between *OPG* A163G, T245G, T950C and G1181C polymorphisms and osteoporosis. There is an obvious mismatch between all previous meta-analyses and this meta-analysis in the subgroup analysis classification. Moreover, after careful reading of the full text, we found that the previous meta-analysis had relevant information and data extraction errors in the process of data extraction, and we corrected them in the extraction process. In addition, the sample size of our study is large. A total of 31 studies were applied in this study (involving 8,402 osteoporosis cases and 7,517 controls), of which 14 studies showed the *OPG* A163G (2,379 cases and 2,229 controls), nine studies investigated the *OPG* T245G (941 cases and 1,019 controls), 12 studies investigated the *OPG* T950C (1,610 cases and 1,234 controls), and 18 studies reported the *OPG* G1181C (3,472 cases and 3,035 controls). In this study, five genetic models were compared separately. Li et al., 2017, however, applied four genetic models; Li et al., 2021, used only three genetic models. At the same time, Li et al., 2017, the ethnicity studied was limited to Chinese. It is important to note that previous meta-analyses did not perform credibility analyses on the results, and all statistically significant relationships were considered “less credible” when we assessed the credibility of previous meta-analyses using the same criteria. Therefore, their research results and conclusions may not be reliable, and they may also be false positive results.

Compared with previous meta-analyses, our study has the following advantages: 1) FPRP, BFDP test and Venice criterion were used for credibility assessment; 2) to evaluate the quality of qualified research; 3) compared with previous meta-analyses, the studies included in this study are more comprehensive, and the data collected are more detailed and accurate; 4) the subgroup analysis was more comprehensive, and it is important to note that not only did we subgroup sex, but we also grouped women separately based on whether they were postmenopausal or not; However, our study still has some potential limitations. First, the current meta-analysis included only published studies, and it is well known that positive results are studied more than negative results. Second, osteoporosis is a complicated polygenic disease, and individual SNPS are slightly associated with osteoporosis risk. However, we have not retrieved data on gene-gene and gene-environment combined effects. Third, although we objectively collected data from the included literature, the control: control rate was less than 1, which may also affect the accuracy of the association. At the same time, there have been relatively few studies of Africans in ethnic analyses and insufficient statistical power to excavate true associations. Therefore, future studies with large samples and sufficiently large subgroups will help validate our findings. Finally, the risk of osteoporosis includes not only genetic factors, but also environmental and lifestyle factors. Our study did not control for various osteoporosis variables, such as smoking, alcohol consumption, breastfeeding, physical activity, estrogen replacement therapy, corticosteroids, calcium supplements et al. In the future, we need to control for various other variables that contribute to osteoporosis, and investigate the association between osteoporosis and genetic, environmental, and lifestyle factors to help validate our findings.

In conclusion, this study indicates that all meaningful results between *OPG* A163G and G1181C polymorphisms and osteoporosis risk were false-positive results rather than true associations. At present, there is still no absolute strong evidence for the real association between *OPG* polymorphism and osteoporosis risk, and further large-scale epidemiological studies are needed to confirm or deny our findings.

## Data Availability

The original contributions presented in the study are included in the article/Supplementary Material, further inquiries can be directed to the corresponding authors.

## Abbreviations

BFDP, Bayesian false discovery probability; CI s, confidence intervals; CNKI, China National Knowledge Infrastructure; FPRP, false-positive report probabilities; HWE, Hardy–Weinberg equilibrium; HWD, Hardy–Weinberg Disequilibrium; ISI, International Statistical Institute; OPG, osteoprotegerin; ORs, odds ratios
