# Supplementary material for: Association between OPG polymorphisms and osteoporosis risk: An updated meta-analysis
Source: Front Genet. 2022 Nov 9;13:1032110. doi: 10.3389/fgene.2022.1032110 (PMC9682267; doi:10.3389/fgene.2022.1032110)
Supplement: Supplementary file 1 [file Table1.docx]

**Supplementary Table 1 Genotype frequencies of *OPG* polymorphism in studies included in this meta-analysis**

| **First author/Year** | **Country** | **Ethnicity** | **Type of controls** | **Sex** | **Female menopause** | **HWE** | | **Number of samples** | | | **Genotypes of cases** | | | **Alleles of cases** | | **Minor allele frequency** | **Genotypes of controls** | | | **Alleles of controls** | | **Minor allele frequency** |
| --- | --- | --- | --- | --- | --- | --- | --- | --- | --- | --- | --- | --- | --- | --- | --- | --- | --- | --- | --- | --- | --- | --- |
|  |  |  |  |  |  | **chi2** | **pr** | **Cases** | **Controls** | **Total** | **A/A** | **A/G** | **G/G** | **A** | **G** |  | **A/A** | **A/G** | **G/G** | **A** | **G** |  |
| **A163G** |  |  |  |  |  |  |  |  |  |  |  |  |  |  |  |  |  |  |  |  |  |  |
| **Langdahl, B.L.et al. 2002** | Denmark | Caucasian | Healthy | Male | NA | 0.021 | 0.886 | 51 | 72 | 123 | 32 | 17 | 2 | 81 | 21 | 0.259 | 51 | 19 | 2 | 121 | 23 | 0.190 |
| **Langdahl, B.L.et al. 2002** | Denmark | Caucasian | Healthy | Female | NP | 0.879 | 0.349 | 215 | 215 | 430 | 143 | 60 | 12 | 346 | 84 | 0.243 | 160 | 49 | 6 | 369 | 61 | 0.165 |
| **Wu, ZZ et al. 2006** | China | Asian | Healthy | Female | P | 2.469 | 0.116 | 73 | 61 | 134 | 12 | 37 | 24 | 61 | 85 | 1.393 | 10 | 22 | 29 | 42 | 80 | 1.905 |
| **Hsu YH et al. 2006** | China | Asian | Healthy | Male | NA | 4.249 | **0.039** | 285 | 290 | 575 | 216 | 65 | 4 | 497 | 73 | 0.147 | 206 | 71 | 13 | 483 | 97 | 0.201 |
| **Geng L et al. 2008** | China | Asian | Healthy | Female | NP | 0.005 | 0.946 | 186 | 214 | 400 | 18 | 66 | 102 | 102 | 270 | 2.647 | 34 | 102 | 78 | 170 | 258 | 1.518 |
| **Seremak-Mrozikiewicz.et al. 2009** | Poland | Caucasian | Healthy | Female | P | 0.090 | 0.764 | 139 | 64 | 203 | 101 | 37 | 1 | 239 | 39 | 0.163 | 51 | 12 | 1 | 114 | 14 | 0.123 |
| **Brambila-Tapia et al. 2012** | Mexico | Mexican-Mestizo | Non- healthy | Female | NP | 0.048 | 0.827 | 9 | 30 | 39 | 5 | 2 | 2 | 12 | 6 | 0.500 | 21 | 8 | 1 | 50 | 10 | 0.200 |
| **Hussien YM et al. 2013** | Egypt | African | Non-healthy | Female | NP | 0.547 | 0.459 | 150 | 50 | 200 | 84 | 42 | 24 | 210 | 90 | 0.429 | 32 | 17 | 1 | 81 | 19 | 0.235 |
| **Bonfa AC et al. 2015** | Brazil | Caucasian | Non-healthy | Female | NP | NA | NA | 51 | 160 | 211 | 32 | 19 | | NA | NA | NA | 92 | 68 | | NA | NA | NA |
| **Boron D et al. 2015** | Poland | Caucasian | Healthy | Female | P | 0.201 | 0.654 | 314 | 63 | 377 | 225 | 84 | 5 | 534 | 94 | 0.176 | 51 | 11 | 1 | 113 | 13 | 0.115 |
| **Selma Cvijetic et al. 2016** | Croatia | Caucasian | Non-healthy | Female | P | 0.284 | 0.594 | 20 | 58 | 78 | 2 | 11 | 7 | 15 | 25 | 1.667 | 1 | 17 | 40 | 19 | 97 | 5.105 |
| **Mydlarova Blascakova et al. 2017** | Slovakia | Caucasian | Healthy | Female | P | 1.044 | 0.307 | 133 | 172 | 305 | 93 | 40 | 0 | 226 | 40 | 0.177 | 134 | 34 | 4 | 302 | 42 | 0.139 |
| **F. Wu et al. 2019** | China | Asian | Healthy | Female | P | 0.503 | 0.478 | 610 | 616 | 1226 | 305 | 245 | 60 | 855 | 365 | 0.427 | 395 | 193 | 28 | 983 | 249 | 0.253 |
| **Abdi, S.et al. 2021** | Saudi Arab | Asian | Healthy | Female | P | 0.839 | 0.360 | 143 | 164 | 307 | 113 | 27 | 3 | 253 | 33 | 0.130 | 127 | 36 | 1 | 290 | 38 | 0.131 |
| **T245G** |  |  |  |  |  |  |  |  |  |  |  |  |  |  |  |  |  |  |  |  |  |  |
| **Langdahl, B.L. et al. 2002** | Denmark | Caucasian | Healthy | Male | NA | 0.093 | 0.760 | 51 | 72 | 123 | 46 | 5 | 0 | 97 | 5 | 0.052 | 67 | 5 | 0 | 139 | 5 | 0.036 |
| **Langdahl, B.L. et al. 2002** | Denmark | Caucasian | Healthy | Female | NP | 0.241 | 0.623 | 216 | 217 | 433 | 188 | 26 | 2 | 402 | 30 | 0.075 | 203 | 14 | 0 | 420 | 14 | 0.033 |
| **Wu, ZZ et al. 2007** | China | Asian | Healthy | Female | P | 2.502 | 0.114 | 73 | 61 | 134 | 5 | 35 | 33 | 45 | 101 | 2.244 | 3 | 31 | 27 | 37 | 85 | 2.297 |
| **Kim, J.G. et al. 2007** | Korea | Asian | Healthy | Female | P | 0.012 | 0.911 | 222 | 163 | 385 | 176 | 46 | 0 | 398 | 46 | 0.116 | 137 | 26 | 0 | 300 | 26 | 0.087 |
| **Dincel, E. et al. 2008** | Turkey | Caucasian | Healthy | Mix | NA | 1.224 | 0.269 | 21 | 21 | 42 | 0 | 3 | 16 | 3 | 35 | 11.667 | 0 | 1 | 20 | 1 | 41 | 41.000 |
| **Mencej-Bedrac. et al. 2011** | Slovenia | Caucasian | Healthy | Female | P | 46.831 | **0.000** | 243 | 245 | 488 | 209 | 32 | 2 | 450 | 36 | 0.080 | 211 | 23 | 11 | 445 | 45 | 0.101 |
| **Bonfa AC et al. 2015** | Brazil | Caucasian | Non- healthy | Female | NP | NA | NA | 51 | 160 | 211 | 35 | 16 | NA | NA | NA | 131 | 29 | NA | NA | NA | NA |  |
| **Zavala-Cerna MG et al.2015** | Mexico | Mexican-Mestizo | Non- healthy | Female | NP | 0.050 | 0.823 | 44 | 22 | 66 | 39 | 4 | 1 | 82 | 6 | 0.073 | 20 | 2 | 0 | 42 | 2 | 0.048 |
| **Selma Cvijetic et al. 2016** | Croatia | Caucasian | Non-healthy | Female | P | NA | NA | 20 | 58 | 78 | 18 | 2 | NA | NA | NA | 51 | 7 | NA | NA | NA |  |  |
| **T950C** |  |  |  |  |  |  |  |  |  |  |  |  |  |  |  |  |  |  |  |  |  |  |
| **Langdahl, B.L. et al. 2002** | Denmark | Caucasian | Healthy | Male | NA | 0.498 | 0.480 | 51 | 72 | 123 | 17 | 25 | 9 | 59 | 43 | 0.729 | 19 | 33 | 20 | 71 | 73 | 1.028 |
| **Langdahl, B.L. et al. 2002** | Denmark | Caucasian | Healthy | Female | NP | 0.530 | 0.467 | 215 | 217 | 432 | 46 | 117 | 52 | 209 | 221 | 1.057 | 53 | 103 | 61 | 209 | 225 | 1.077 |
| **Wu ZZ. et al. 2005** | China | Asian | Healthy | Female | P | 1.599 | 0.206 | 73 | 61 | 134 | 28 | 39 | 6 | 95 | 51 | 0.537 | 29 | 29 | 3 | 87 | 35 | 0.402 |
| **Vidal C et al. 2006** | Malta | Caucasian | Healthy | Female | P | 3.623 | 0.057 | 181 | 119 | 300 | 83 | 57 | 41 | 223 | 139 | 0.623 | 17 | 43 | 59 | 77 | 161 | 2.091 |
| **Sui MM. et al. 2008** | China | Asian | Healthy | Mix | NA | 0.166 | 0.683 | 272 | 208 | 480 | 75 | 132 | 65 | 282 | 262 | 0.929 | 56 | 101 | 51 | 213 | 203 | 0.953 |
| **Geng L et al. 2008** | China | Asian | Healthy | Female | NP | 0.466 | 0.495 | 186 | 214 | 400 | 70 | 76 | 40 | 216 | 156 | 0.722 | 72 | 100 | 42 | 244 | 184 | 0.754 |
| **Li XR et al. 2009** | China | Asian | Healthy | Male | NA | 0.012 | 0.914 | 98 | 101 | 199 | 25 | 39 | 34 | 89 | 107 | 1.202 | 33 | 49 | 19 | 115 | 87 | 0.757 |
| **Liu JM et al. 2010** | China | Asian | Non-healthy | Female | P | 0.809 | 0.368 | 50 | 50 | 100 | 18 | 23 | 9 | 59 | 41 | 0.695 | 9 | 28 | 13 | 46 | 54 | 1.174 |
| **Tao YH et al. 2011** | China | Asian | Healthy | Female | P | 2.567 | 0.109 | 77 | 54 | 131 | 22 | 45 | 10 | 89 | 65 | 0.730 | 20 | 30 | 4 | 70 | 38 | 0.543 |
| **Zavala-Cerna MG et al. 2015** | Mexico | Mexican-Mestizo | Non-healthy | Female | NP | 0.126 | 0.723 | 36 | 18 | 54 | 6 | 14 | 16 | 26 | 46 | 1.769 | 2 | 7 | 9 | 11 | 25 | 2.273 |
| **Boron D et al. 2015** | Poland | Caucasian | Healthy | Female | NP | 0.151 | 0.698 | 306 | 59 | 365 | 76 | 147 | 83 | 299 | 313 | 1.047 | 15 | 28 | 16 | 58 | 60 | 1.034 |
| **LI Boyi et al. 2022** | China | Asian | Non-healthy | Mix | NA | 0.599 | 0.439 | 65 | 61 | 126 | 13 | 36 | 16 | 62 | 68 | 1.097 | 21 | 32 | 8 | 74 | 48 | 0.649 |
| **G1181C** |  |  |  |  |  |  |  |  |  |  |  |  |  |  |  |  |  |  |  |  |  |  |
| **Langdahl, B.L. et al. 2002** | Denmark | Caucasian | Healthy | Male | NA | 1.795 | 0.180 | 50 | 72 | 122 | 11 | 30 | 9 | 52 | 48 | 0.923 | 14 | 29 | 29 | 57 | 87 | 1.526 |
| **Langdahl, B.L. et al. 2002** | Denmark | Caucasian | Healthy | Female | NP | 2.607 | 0.106 | 216 | 217 | 433 | 37 | 118 | 61 | 192 | 240 | 1.250 | 47 | 95 | 75 | 189 | 245 | 1.296 |
| **Zhao, H.Y. et al. 2005** | China | Asian | Healthy | Female | P | 0.011 | 0.918 | 134 | 71 | 205 | 85 | 40 | 9 | 210 | 58 | 0.276 | 30 | 32 | 9 | 92 | 50 | 0.543 |
| **Hsu YH et al. 2006** | China | Asian | Healthy | Female | NP | 3.097 | 0.078 | 285 | 290 | 575 | 175 | 93 | 17 | 443 | 127 | 0.287 | 163 | 101 | 26 | 427 | 153 | 0.358 |
| **Vidal C et al. 2006** | Malta | Caucasian | Healthy | Female | P | 2.094 | 0.148 | 181 | 119 | 300 | 76 | 50 | 55 | 202 | 160 | 0.792 | 24 | 50 | 45 | 98 | 140 | 1.429 |
| **Kim, J.G. et al. 2007** | Korea | Asian | Healthy | Female | P | 3.765 | 0.052 | 222 | 163 | 385 | 133 | 74 | 15 | 340 | 104 | 0.306 | 89 | 56 | 18 | 234 | 92 | 0.393 |
| **Geng L et al. 2008** | China | Asian | Healthy | Female | NP | 1.834 | 0.176 | 186 | 214 | 400 | 94 | 75 | 17 | 263 | 109 | 0.414 | 98 | 87 | 29 | 283 | 145 | 0.512 |
| **Mencej-Bedrac. et al. 2009** | Slovenia | Asian | Healthy | Female | P | 0.025 | 0.874 | 239 | 228 | 467 | 59 | 117 | 63 | 235 | 243 | 1.034 | 39 | 112 | 77 | 190 | 266 | 1.400 |
| **Seremak-Mrozikiewicz. et al. 2009** | Poland | Caucasian | Healthy | Female | P | 0.331 | 0.565 | 139 | 64 | 203 | 27 | 73 | 39 | 127 | 151 | 1.189 | 12 | 34 | 18 | 58 | 70 | 1.207 |
| **Tao YH et al. 2011** | China | Asian | Healthy | Female | P | 0.196 | 0.658 | 71 | 20 | 91 | 50 | 19 | 2 | 119 | 23 | 0.193 | 8 | 10 | 2 | 26 | 14 | 0.538 |
| **Mencej-Bedrac. et al. 2011** | Slovenia | Caucasian | Healthy | Female | P | 0.206 | 0.650 | 243 | 235 | 478 | 61 | 118 | 64 | 240 | 246 | 1.025 | 59 | 114 | 62 | 232 | 238 | 1.026 |
| **Bonfa AC et al. 2015** | Brazil | Caucasian | Non- healthy | Female | NP | NA | NA | 51 | 160 | 211 | NA | NA | | NA | NA | NA | 57 | 103 | | NA | NA | NA |
| **Boron D et al. 2015** | Poland | Caucasian | Healthy | Female | P | 0.051 | 0.821 | 314 | 64 | 378 | 57 | 171 | 86 | 285 | 343 | 1.204 | 11 | 30 | 23 | 52 | 76 | 1.462 |
| **Nava-Valdivia et al. 2017** | Mexico | Mexican-Mestizo | Non- healthy | Female | NP | 0.347 | 0.556 | 131 | 45 | 176 | 93 | 34 | 4 | 220 | 42 | 0.191 | 29 | 15 | 1 | 73 | 17 | 0.233 |
| **F. Wu et al. 2019** | China | Asian | Healthy | Female | P | 0.006 | 0.939 | 610 | 616 | 1226 | 339 | 226 | 45 | 904 | 316 | 0.350 | 362 | 220 | 34 | 944 | 288 | 0.305 |
| **González-Mercado et al. 2019** | Mexico | Mexican-Mestizo | Healthy | Female | P | 1.268 | 0.260 | 87 | 87 | 174 | 21 | 37 | 29 | 79 | 95 | 1.203 | 21 | 38 | 28 | 80 | 94 | 1.175 |
| **Abdi, S. et al. 2021** | Saudi Arab | Asian | Healthy | Female | P | 0.553 | 0.457 | 174 | 198 | 372 | 23 | 64 | 87 | 110 | 238 | 2.164 | 20 | 93 | 85 | 133 | 263 | 1.977 |
| **Mydlarova Blascakova et al. 2021** | Slovak | Caucasian | Healthy | Female | P | 0.586 | 0.444 | 139 | 172 | 311 | 27 | 64 | 48 | 118 | 160 | 1.356 | 34 | 87 | 51 | 155 | 189 | 1.219 |

HWE = Hardy–Weinberg equilibrium； NA = not available；P = Postmenopausal women；NP = Non-postmenopausal women
