# Supplementary material for: Association between OPG polymorphisms and osteoporosis risk: An updated meta-analysis
Source: Front Genet. 2022 Nov 9;13:1032110. doi: 10.3389/fgene.2022.1032110 (PMC9682267; doi:10.3389/fgene.2022.1032110)
Supplement: Supplementary file 2 [file Table2.docx]

**Supplementary Table 2 Main characteristics and Quality score of studies included**

| **First author / Year** | **Country** | **Ethnicity** | **Sex** | **Female menopause** | **Polymorphism genotyping technique** | **Cases** | | | | **Controls** | | | | **Quality score** |
| --- | --- | --- | --- | --- | --- | --- | --- | --- | --- | --- | --- | --- | --- | --- |
|  |  |  |  |  |  | **N** | **Age1** | **Diagnosis** | **Matching** | **N** | **Age1** | **HWE** | **Type** |  |
| **A163G** |  |  |  |  |  |  |  |  |  |  |  |  |  |  |
| **Langdahl, B.L.et al. 2002** | Denmark | Caucasian | Male | NA | PCR-RFLP | 51 | 56.0±13.6 | Not described | Age and sex | 72 | 51.2±15.9 | 0.886 | Healthy | 13 |
| **Langdahl, B.L.et al. 2002** | Denmark | Caucasian | Female | Non-postmenopausal women | PCR-RFLP | 215 | 64.2 ± 9.2 | Not described | sex | 215 | 57.8±15.2 | 0.349 | Healthy | 13 |
| **Wu, ZZ et al. 2006** | China | Asian | Female | Postmenopausal women | PCR-RFLP | 73 | 64.6 | WHO | sex | 61 | 60.7 | 0.116 | Healthy | 16 |
| **Hsu YH et al. 2006** | China | Asian | Male | NA | TaqMan | 285 | 47.8±7.1 | WHO | Age and sex | 290 | 47.7±7.2 | **0.039** | Healthy | 16 |
| **Geng L et al. 2008** | China | Asian | Female | Non-postmenopausal women | PCR-RFLP | 186 | NR | WHO | Age and sex | 214 | NR | 0.946 | Healthy | 13 |
| **Seremak-Mrozikiewicz.et al. 2009** | Poland | Caucasian | Female | Postmenopausal women | PCR-RFLP | 139 | 56.06±8.83 | WHO | Age and sex | 64 | 53.38±8.22 | 0.764 | Healthy | 13 |
| **Brambila-Tapia et al. 2012** | Mexico | Mexican-Mestizo | Female | Non-postmenopausal women | PCR-RFLP | 9 | 49.3±4.61 | WHO | Age and sex | 30 | 48.5± 6.13 | 0.827 | Non-healthy | 11 |
| **Hussien YM et al. 2013** | Egypt | African | Female | Non-postmenopausal women | PCR-RFLP | 150 | NR | WHO | Age and sex | 50 | NR | 0.459 | Non-healthy | 12 |
| **Bonfa AC et al. 2015** | Brazil | Caucasian | Female | Non-postmenopausal women | TaqMan | 51 | NR | Z-score≤−2.0 | sex | 160 | NR | NA | Non-healthy | 13 |
| **Boron D et al. 2015** | Poland | Caucasian | Female | Postmenopausal women | RT-PCR | 314 | NR | Not described | sex | 63 | NR | 0.654 | Healthy | 11 |
| **Selma Cvijetic et al. 2016** | Croatia | Caucasian | Female | Postmenopausal women | PCR-RFLP | 20 | 65.6±12.6 | WHO | Age and sex | 58 | 60.8 ±8.7 | 0.594 | Non-healthy | 11 |
| **Mydlarova Blascakova et al. 2017** | Slovakia | Caucasian | Female | Postmenopausal women | TaqMan | 133 | 57.53±9.15 | WHO | Age and sex | 172 | 58.00±5.79 | 0.307 | Healthy | 16 |
| **F. Wu et al. 2019** | China | Asian | Female | Postmenopausal women | PCR-RFLP | 610 | 63.02±9.53 | WHO | Age and sex | 616 | 54.37±7.45 | 0.478 | Healthy | 14 |
| **Abdi, S.et al. 2021** | Saudi Arab | Asian | Female | Postmenopausal women | TaqMan | 143 | 73.8±18.8 | WHO | sex | 164 | 72.6±19.7 | 0.360 | Healthy | 15 |
| **T245G** |  |  |  |  |  |  |  |  |  |  |  |  |  |  |
| **Langdahl, B.L. et al. 2002** | Denmark | Caucasian | Male | NA | PCR-RFLP | 51 | 56.0±13.6 | Not described | Age and sex | 72 | 51.2 ± 15.9 | 0.760 | Healthy | 13 |
| **Langdahl, B.L. et al. 2002** | Denmark | Caucasian | Female | Non-postmenopausal women | PCR-RFLP | 216 | 64.2 ± 9.2 | Not described | sex | 217 | 57.8 ± 15.2 | 0.623 | Healthy | 13 |
| **Wu, ZZ et al. 2007** | China | Asian | Female | Postmenopausal women | PCR-RFLP | 73 | 64.59 | WHO | Age and sex | 61 | 60.66 | 0.114 | Healthy | 16 |
| **Kim, J.G. et al. 2007** | Korea | Asian | Female | Postmenopausal women | PCR-RFLP | 222 | NR | WHO | sex | 163 | NR | 0.911 | Healthy | 13 |
| **Dincel, E. et al. 2008** | Turkey | Caucasian | Mix | NA | PCR-RFLP | 21 | 74.47± 8.91 | Not described | sex | 21 | 75.47 ± 7.44 | 0.269 | Healthy | 10 |
| **Mencej-Bedrac. et al. 2011** | Slovenia | Caucasian | Female | Postmenopausal women | PCR-RFLP | 243 | 61.5 ± 8.3 | WHO | Age and sex | 245 | 64.4 ± 8.2 | **0.000** | Healthy | 12 |
| **Bonfa AC et al. 2015** | Brazil | Caucasian | Female | Non-postmenopausal women | TaqMan | 51 | NR | Z-score≤−2.0 | sex | 160 | NR | NA | Non-healthy | 13 |
| **Zavala-Cerna MG et al.2015** | Mexico | Mexican-Mestizo | Female | Non-postmenopausal women | PCR-RFLP | 44 | NR | WHO | sex | 22 | NR | 0.823 | Non-healthy | 13 |
| **Selma Cvijetic et al. 2016** | Croatia | Caucasian | Female | Postmenopausal women | PCR-RFLP | 20 | 65.6 ±12.6 | WHO | Age and sex | 58 | 60.8 ±8.7 | NA | Non-healthy | 11 |
| **T950C** |  |  |  |  |  |  |  |  |  |  |  |  |  |  |
| **Langdahl, B.L. et al. 2002** | Denmark | Caucasian | Male | NA | PCR-RFLP | 51 | 56.0±13.6 | Not described | Age and sex | 72 | 51.2 ± 15.9 | 0.480 | Healthy | 13 |
| **Langdahl, B.L. et al. 2002** | Denmark | Caucasian | Female | Non-postmenopausal women | PCR-RFLP | 215 | 64.2 ± 9.2 | Not described | sex | 217 | 57.8 ± 15.2 | 0.467 | Healthy | 13 |
| **Wu ZZ. et al. 2005** | China | Asian | Female | Postmenopausal women | PCR-RFLP | 73 | 64.59 | WHO | Age and sex | 61 | 60.66 | 0.206 | Healthy | 16 |
| **Vidal C et al. 2006** | Malta | Caucasian | Female | Postmenopausal women | PCR-RFLP | 181 | NR | WHO | sex | 119 | NR | 0.057 | Healthy | 13 |
| **Sui MM. et al. 2008** | China | Asian | Mix | NA | PCR-RFLP | 272 | NR | Not described | NR | 208 | NR | 0.683 | Healthy | 8 |
| **Geng L et al. 2008** | China | Asian | Female | Non-postmenopausal women | PCR-RFLP | 186 | NR | WHO | sex | 214 | NR | 0.495 | Healthy | 13 |
| **Li XR et al. 2009** | China | Asian | Male | NA | PCR-RFLP | 98 | 70.9±8.2 | T-score≤-2.0 | Age and sex | 101 | 71.0 ± 7.5 | 0.914 | Healthy | 11 |
| **Liu JM et al. 2010** | China | Asian | Female | Postmenopausal women | PCR-RFLP | 50 | NR | WHO | sex | 50 | NR | 0.368 | Non-healthy | 10 |
| **Tao YH et al. 2011** | China | Asian | Female | Postmenopausal women | AllGlo | 77 | 57.08±3.19 | WHO | Age and sex | 54 | 56.74±2.69 | 0.109 | Healthy | 11 |
| **Zavala-Cerna MG et al. 2015** | Mexico | Mexican-Mestizo | Female | Non-postmenopausal women | PCR-RFLP | 36 | NR | WHO | sex | 18 | NR | 0.723 | Non-healthy | 13 |
| **Boron D et al. 2015** | Poland | Caucasian | Female | Non-postmenopausal women | RT-PCR | 306 | NR | Not described | sex | 59 | NR | 0.698 | Healthy | 11 |
| **LI Boyi et al. 2022** | China | Asian | Mix | NA | PCR-RFLP | 65 | 66.33±6.77 | WHO | Age and sex | 61 | 61.65 ± 8.16 | 0.439 | Non-healthy | 13 |
| **G1181C** |  |  |  |  |  |  |  |  |  |  |  |  |  |  |
| **Langdahl, B.L. et al. 2002** | Denmark | Caucasian | Male | NA | PCR-RFLP | 50 | 56.0±13.6 | Not described | Age and sex | 72 | 51.2 ± 15.9 | 0.180 | Healthy | 13 |
| **Langdahl, B.L. et al. 2002** | Denmark | Caucasian | Female | Non-postmenopausal women | PCR-RFLP | 216 | 64.2 ± 9.2 | Not described | sex | 217 | 57.8 ± 15.2 | 0.106 | Healthy | 13 |
| **Zhao, H.Y. et al. 2005** | China | Asian | Female | Postmenopausal women | Sequencing | 134 | NR | WHO | Age and sex | 71 | NR | 0.918 | Healthy | 12 |
| **Hsu YH et al. 2006** | China | Asian | Female | Non-postmenopausal women | TaqMan | 285 | 63.0±0.53 | WHO | Age and sex | 290 | 61.4±0.71 | 0.078 | Healthy | 18 |
| **Vidal C et al. 2006** | Malta | Caucasian | Female | Postmenopausal women | PCR-RFLP | 181 | NR | WHO | sex | 119 | NR | 0.148 | Healthy | 13 |
| **Kim, J.G. et al. 2007** | Korea | Asian | Female | Postmenopausal women | PCR-RFLP | 222 | NR | WHO | sex | 163 | NR | 0.052 | Healthy | 13 |
| **Geng L et al. 2008** | China | Asian | Female | Non-postmenopausal women | PCR-RFLP | 186 | NR | WHO | Age and sex | 214 | NR | 0.176 | Healthy | 13 |
| **Mencej-Bedrac. et al. 2009** | Slovenia | Asian | Female | Postmenopausal women | TaqMan | 239 | 64.5±8.2 | WHO | Age and sex | 228 | 61.5±8.3 | 0.874 | Healthy | 13 |
| **Seremak-Mrozikiewicz. et al. 2009** | Poland | Caucasian | Female | Postmenopausal women | PCR-RFLP | 139 | 56.06±8.83 | WHO | Age and sex | 64 | 53.38±8.22 | 0.565 | Healthy | 13 |
| **Tao YH et al. 2011** | China | Asian | Female | Postmenopausal women | AllGlo | 71 | 57.08±3.19 | WHO | Age and sex | 20 | 56.74±2.69 | 0.658 | Healthy | 11 |
| **Mencej-Bedrac. et al. 2011** | Slovenia | Caucasian | Female | Postmenopausal women | PCR-RFLP | 243 | 61.5 ± 8.3 | WHO | Age and sex | 235 | 64.4 ± 8.2 | 0.650 | Healthy | 12 |
| **Bonfa AC et al. 2015** | Brazil | Caucasian | Female | Non-postmenopausal women | TaqMan | 51 | NR | Z-score≤−2.0 | sex | 160 | NR | NA | Non-healthy | 13 |
| **Boron D et al. 2015** | Poland | Caucasian | Female | Postmenopausal women | RT-PCR | 314 | NR | Not described | sex | 64 | NR | 0.821 | Healthy | 11 |
| **Nava-Valdivia et al. 2017** | Mexico | Mexican-Mestizo | Female | Non-postmenopausal women | PCR-RFLP | 131 | 62 ±9 | WHO | Age and sex | 45 | 54 ± 7 | 0.556 | Non-healthy | 12 |
| **F. Wu et al. 2019** | China | Asian | Female | Postmenopausal women | PCR-RFLP | 610 | 73.8 ± 18.8 | WHO | Age and sex | 616 | 72.6 ± 19.7 | 0.939 | Healthy | 14 |
| **González-Mercado et al. 2019** | Mexico | Mexican-Mestizo | Female | Postmenopausal women | Sequencing | 87 | NR | WHO | sex | 87 | NR | 0.260 | Healthy | 12 |
| **Abdi, S. et al. 2021** | Saudi Arab | Asian | Female | Postmenopausal women | TaqMan | 174 | 58.7 ± 7.8 | WHO | sex | 198 | 53.4 ± 5.9 | 0.457 | Healthy | 15 |
| **Mydlarova Blascakova et al. 2021** | Slovak | Caucasian | Female | Postmenopausal women | TaqMan | 139 | NR | WHO | Age and sex | 172 | 55.68 ± 9.40 | 0.444 | Healthy | 14 |

1= (Mean±SD)yrs；HWE = Hardy–Weinberg equilibrium；NR = not reported; NA = not available; PCR-RFLP, polymerase chain reaction–restriction fragment length polymorphism; NR, not reported; RT-PCR, real-time polymerase chain reaction.
