# Supplementary material for: Association between OPG polymorphisms and osteoporosis risk: An updated meta-analysis
Source: Front Genet. 2022 Nov 9;13:1032110. doi: 10.3389/fgene.2022.1032110 (PMC9682267; doi:10.3389/fgene.2022.1032110)
Supplement: Supplementary file 4 [file Table3.docx]

Supplementary Table 3. Scale for quality assessment of molecular association studies

| Criterion | Score |
| --- | --- |
| Source of case  Selected from population | 2 |
| Selected from hospital | 1 |
| Not described | 0 |
| Source of control  Population-based | 3 |
| Blood donors or volunteers | 2 |
| Hospital-based | 1 |
| Not described | 0 |
| Ascertainment of osteoporosis  WHO | 2 |
| Diagnosis of osteoporosis by patient medical record | 1 |
| Not described | 0 |
| Ascertainment of control  Controls were tested to screen out | 2 |
| Controls were subjects who did not report osteoporosis, no objective testing | 1 |
| Not described | 0 |
| Matching  Controls matched with cases by age and sex | 2 |
| Controls matched with cases only by age or sex | 1 |
| Not matched or not described | 0 |
| Genotyping examination  Genotyping done blindly and quality control | 2 |
| Only genotyping done blindly or quality control | 1 |
| Unblinded and without quality control | 0 |
| Specimens used for determining genotypes  Blood cells or normal tissues | 1 |
| Tumor tissues or exfoliated cells of tissue | 0 |
| HWE  HWE in the control group | 1 |
| Hardy-Weinberg disequilibrium in the control group | 0 |
| Association assessment  Assess association between genotypes and osteoporosis with appropriate statistics and adjustment for confounders | 2 |
| Assess association between genotypes and osteoporosis with appropriate statistics without adjustment for confounders | 1 |
| Inappropriate statistics used | 0 |
| Total sample size  >500 | 3 |
| 200-500 | 2 |
| <200  HWE: Hardy-Weinberg equilibrium | 1 |
