# Supplementary material for: Association between OPG polymorphisms and osteoporosis risk: An updated meta-analysis
Source: Front Genet. 2022 Nov 9;13:1032110. doi: 10.3389/fgene.2022.1032110 (PMC9682267; doi:10.3389/fgene.2022.1032110)
Supplement: Supplementary file 5 [file Table4.docx]

**Supplementary Table 4. Quality assessment of included studies for assessing the quality of case control studies.**

| **First**  **Author/Year** | **Criterion** | | | | | | | | | | **Quality**  **Score**  **(Total**  **score)** |
| --- | --- | --- | --- | --- | --- | --- | --- | --- | --- | --- | --- |
|  | **Source of case** | **Source of control** | **Ascertainment of osteoporosis** | **Ascertainment of control** | **Matching** | **Genotyping examination** | **Specimens used for determining genotypes** | **HWE** | **Association assessment** | **Total sample size** |  |
| **A163G** | | | | | | | | | | | |
| **Langdahl, B.L. et al., 2002** | 1 | 3 | 0 | 1 | 2 | 1 | 1 | 1 | 2 | 1 | **13** |
| **Langdahl, B.L. et al., 2002** | 1 | 3 | 0 | 1 | 1 | 1 | 1 | 1 | 2 | 2 | **13** |
| **Wu Z.Z et al., 2006** | 2 | 3 | 2 | 2 | 1 | 1 | 1 | 1 | 2 | 1 | **16** |
| **Hsu Y.H et al., 2006** | 2 | 3 | 2 | 2 | 2 | 0 | 1 | 0 | 1 | 3 | **16** |
| **Geng L et al., 2008** | 1 | 1 | 2 | 0 | 2 | 1 | 1 | 1 | 2 | 2 | **13** |
| **Seremak-Mrozikiewicz. et al., 2009** | 1 | 1 | 2 | 2 | 2 | 1 | 1 | 1 | 0 | 2 | **13** |
| **Brambila-Tapia et al., 2012** | 1 | 1 | 2 | 2 | 2 | 0 | 1 | 1 | 0 | 1 | **11** |
| **Hussien YM et al., 2013** | 1 | 1 | 2 | 2 | 2 | 0 | 1 | 1 | 0 | 2 | **12** |
| **Bonfa AC et al., 2015** | 1 | 1 | 0 | 2 | 1 | 2 | 1 | 1 | 2 | 2 | **13** |
| **Boron D et al., 2015** | 2 | 3 | 0 | 2 | 1 | 0 | 0 | 1 | 0 | 2 | **11** |
| **Selma Cvijetic et al., 2016** | 1 | 1 | 2 | 2 | 2 | 0 | 1 | 1 | 0 | 1 | **11** |
| **Mydlarova Blascakova et al., 2017** | 2 | 3 | 2 | 2 | 2 | 0 | 1 | 1 | 2 | 1 | **16** |
| **Wu F. et al., 2019** | 1 | 2 | 2 | 2 | 2 | 0 | 1 | 1 | 0 | 3 | **14** |
| **Abdi, S. et al., 2021** | 1 | 1 | 2 | 2 | 1 | 2 | 1 | 1 | 2 | 2 | **15** |
| **T245G** | | | | | | | | | | | |
| **Langdahl, B.L. et al., 2002** | 1 | 3 | 0 | 1 | 2 | 1 | 1 | 1 | 2 | 1 | **13** |
| **Langdahl, B.L. et al., 2002** | 1 | 3 | 0 | 1 | 1 | 1 | 1 | 1 | 2 | 2 | **13** |
| **Wu Z.Z et al., 2007** | 2 | 3 | 2 | 2 | 2 | 1 | 1 | 1 | 1 | 1 | **16** |
| **Kim J.G. et al., 2007** | 1 | 1 | 2 | 2 | 1 | 0 | 1 | 1 | 2 | 2 | **13** |
| **Dincel, E et al., 2008** | 1 | 2 | 0 | 2 | 1 | 0 | 1 | 1 | 1 | 1 | **10** |
| **Mencej-Bedrac. et al., 2011** | 1 | 1 | 2 | 2 | 2 | 0 | 1 | 1 | 0 | 2 | **12** |
| **Bonfa AC et al., 2015** | 1 | 1 | 0 | 2 | 1 | 2 | 1 | 1 | 2 | 2 | **13** |
| **Zavala-Cerna MG et al., 2015** | 1 | 2 | 2 | 2 | 1 | 1 | 1 | 1 | 1 | 1 | **13** |
| **Selma Cvijetic et al., 2016** | 1 | 1 | 2 | 2 | 2 | 0 | 1 | 1 | 0 | 1 | **11** |
| **T950C** | | | | | | | | | | | |
| **Langdahl, B.L. et al., 2002** | 1 | 3 | 0 | 1 | 2 | 1 | 1 | 1 | 2 | 1 | **13** |
| **Langdahl, B.L. et al., 2002** | 1 | 3 | 0 | 1 | 1 | 1 | 1 | 1 | 2 | 2 | **13** |
| **Wu Z.Z.et al., 2005** | 2 | 3 | 2 | 2 | 2 | 1 | 1 | 1 | 1 | 1 | **16** |
| **Vidal C et al., 2006** | 1 | 1 | 2 | 2 | 1 | 1 | 1 | 1 | 1 | 2 | **13** |
| **Sui M.M. et al., 2008** | 1 | 1 | 0 | 2 | 0 | 0 | 1 | 1 | 0 | 2 | **8** |
| **Geng L et al., 2008** | 1 | 1 | 2 | 0 | 2 | 1 | 1 | 1 | 2 | 2 | **13** |
| **Li X.R et al., 2009** | 1 | 3 | 0 | 2 | 2 | 0 | 1 | 1 | 0 | 1 | **11** |
| **Liu J.M et al., 2010** | 1 | 1 | 2 | 2 | 1 | 0 | 1 | 1 | 0 | 1 | **10** |
| **Tao Y.H et al., 2011** | 1 | 1 | 2 | 2 | 2 | 0 | 1 | 1 | 0 | 1 | **11** |
| **Zavala-Cerna MG et al., 2015** | 1 | 2 | 2 | 2 | 1 | 1 | 1 | 1 | 1 | 1 | **13** |
| **Boron D et al., 2015** | 2 | 3 | 0 | 2 | 1 | 0 | 0 | 1 | 0 | 2 | **11** |
| **LI B.Y. et al., 2022** | 1 | 1 | 2 | 2 | 2 | 1 | 1 | 1 | 1 | 1 | **13** |
| **G1181C** | | | | | | | | | | | |
| **Langdahl, B.L. et al., 2002** | 1 | 3 | 0 | 1 | 2 | 1 | 1 | 1 | 2 | 1 | **13** |
| **Langdahl, B.L. et al., 2002** | 1 | 3 | 0 | 1 | 1 | 1 | 1 | 1 | 2 | 2 | **13** |
| **Zhao H.Y. et al., 2005** | 0 | 0 | 2 | 2 | 2 | 1 | 1 | 1 | 1 | 2 | **12** |
| **Hsu Y.H et al., 2006** | 2 | 3 | 2 | 2 | 2 | 0 | 1 | 1 | 2 | 3 | **18** |
| **Vidal C et al., 2006** | 1 | 1 | 2 | 2 | 1 | 1 | 1 | 1 | 1 | 2 | **13** |
| **Kim J.G. et al., 2007** | 1 | 1 | 2 | 2 | 1 | 0 | 1 | 1 | 2 | 2 | **13** |
| **Geng L et al., 2008** | 1 | 1 | 2 | 0 | 2 | 1 | 1 | 1 | 2 | 2 | **13** |
| **Mencej-Bedrac. et al., 2009** | 1 | 1 | 2 | 2 | 2 | 0 | 1 | 1 | 1 | 2 | **13** |
| **Seremak-Mrozikiewicz. et al., 2009** | 1 | 1 | 2 | 2 | 2 | 1 | 1 | 1 | 0 | 2 | **13** |
| **Tao Y.H et al., 2011** | 1 | 1 | 2 | 2 | 2 | 0 | 1 | 1 | 0 | 1 | **11** |
| **Mencej-Bedrac. et al., 2011** | 1 | 1 | 2 | 2 | 2 | 0 | 1 | 1 | 0 | 2 | **12** |
| **Bonfa AC et al., 2015** | 1 | 1 | 0 | 2 | 1 | 2 | 1 | 1 | 2 | 2 | **13** |
| **Boron D et al., 2015** | 2 | 3 | 0 | 2 | 1 | 0 | 0 | 1 | 0 | 2 | **11** |
| **Nava-Valdivia et al., 2017** | 1 | 1 | 2 | 2 | 2 | 0 | 1 | 1 | 1 | 1 | **12** |
| **Wu F. et al., 2019** | 1 | 2 | 2 | 2 | 2 | 0 | 1 | 1 | 0 | 3 | **14** |
| **González-Mercado et al., 2019** | 1 | 1 | 2 | 2 | 1 | 1 | 1 | 1 | 1 | 1 | **12** |
| **Abdi, S. et al., 2021** | 1 | 1 | 2 | 2 | 1 | 2 | 1 | 1 | 2 | 2 | **15** |
| **Mydlarova Blascakova et al., 2021** | 2 | 3 | 2 | 2 | 2 | 0 | 1 | 1 | 0 | 1 | **14** |

HWE, Hardy–Weinberg equilibrium
